# **Development of an Affinity-Based Probe to Profile Endogenous Human Adenosine A_3_ Receptor Expression**

**DOI:** 10.1021/acs.jmedchem.3c00854

**Published:** 2023-08-02

**Authors:** Bert L. H. Beerkens, Inge M. Snijders, Joep Snoeck, Rongfang Liu, Anton T. J. Tool, Sylvia E. Le Dévédec, Willem Jespers, Taco W. Kuijpers, Gerard J.P. van Westen, Laura H. Heitman, Adriaan P. IJzerman, Daan van der Es

**Affiliations:** † Division of Drug Discovery and Safety, Leiden Academic Centre for Drug Research, Leiden University, Einsteinweg 55, 2333 CC Leiden, The Netherlands; ‡ Department of Molecular Hematology, Sanquin Research, Plesmalaan 125, 1066 CX Amsterdam, The Netherlands; § Department of Pediatric Immunology, Rheumatology and Infectious Diseases, Emma Children’s Hospital, Academic Medical Center, University of Amsterdam, Amsterdam, The Netherlands, Meibergdreef 9, 1105 AZ Amsterdam, The Netherlands; ∥ Oncode Institute, Einsteinweg 55, 2333 CC Leiden, The Netherlands

## Abstract

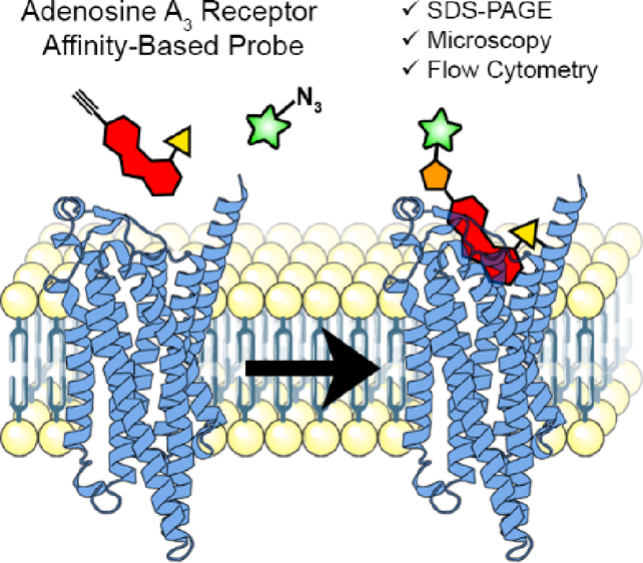

The adenosine A_3_ receptor (A_3_AR) is a G protein-coupled receptor (GPCR) that exerts immunomodulatory effects in pathophysiological conditions such as inflammation and cancer. Thus far, studies toward the downstream effects of A_3_AR activation have yielded contradictory results, thereby motivating the need for further investigations. Various chemical and biological tools have been developed for this purpose, ranging from fluorescent ligands to antibodies. Nevertheless, these probes are limited by their reversible mode of binding, relatively large size, and often low specificity. Therefore, in this work, we have developed a clickable and covalent affinity-based probe (AfBP) to target the human A_3_AR. Herein, we show validation of the synthesized AfBP in radioligand displacement, SDS-PAGE, and confocal microscopy experiments as well as utilization of the AfBP for the detection of endogenous A_3_AR expression in flow cytometry experiments. Ultimately, this AfBP will aid future studies toward the expression and function of the A_3_AR in pathologies.

## Introduction

Adenosine is a signaling molecule that is the endogenous agonist to four adenosine receptors (ARs): the A_1_, A_2A_, A_2B_, and A_3_ adenosine receptors (A_1_AR, A_2A_AR, A_2B_AR, and A_3_AR) that are members of the larger G protein-coupled receptor (GPCR) family.^1−3^ Activation of the ARs via binding of adenosine induces a cascade of intracellular signaling pathways that in turn modulate the cellular response to physiological and pathophysiological conditions, examples being inflammation, autoimmune disorders, and cancers.^4−6^

The ARs are expressed on diverse cell and tissue types, in which the receptors all exert their own functions.^1^ In the case of the human A_3_AR (hA_3_AR), the receptor has been found expressed on granulocytes: eosinophils, neutrophils, and mast cells among other cell types.^7−10^ Here, activation of the hA_3_AR leads to various immunomodulatory effects, ranging from degranulation to influencing chemotaxis.^7−13^ However, multiple contradictory observations have been reported regarding the activation of hA_3_ARs. For example, both inhibition and promotion of chemotaxis have been observed upon addition of a selective hA_3_AR agonist to neutrophils.^11,13,14^ Next to that, expression of the hA_3_AR is species-dependent, and large differences in hA_3_AR activity have been found between humans and rodents.^15,16^ Thus, many questions regarding activity and functioning of the hA_3_AR, whether on granulocytes or on other cell types and tissues, remain unanswered.

Most of the aforementioned studies have been carried out using selective ligands, e.g., agonists or antagonists to induce a cellular response as read-out. This has yielded valuable information on a biological level but ignores multiple factors that influence receptor signaling on a molecular level, such as receptor localization, protein–protein interactions (PPIs) and post-translational modifications (PTMs).^17^ Traditionally, these aspects would be studied using antibodies. However, antibodies for GPCRs are hindered in their selectivity due to the low expression levels of GPCRs and the high conformational variability of extracellular epitopes on GPCRs.^18^ This is especially true for the ARs that are lacking an extended extracellular N-terminus.

In the past decade, multiple small molecules have been developed as tool compounds to study the hA_3_AR on a molecular level. Most prominently developed are the fluorescent ligands: agonists or antagonists conjugated to a fluorophore.^19−28^ Noteworthy, one of these fluorescent ligands has been used to study internalization, localization and certain PPIs of the hA_3_AR on hA_3_AR-overexpressing Chinese hamster ovary (CHO) cells as well as activated neutrophils.^12,24^ Yet, the current use of fluorescent ligands is limited to the type of fluorophore conjugated, a fluorescent read-out in specific assay types, and reversible binding to the receptor. Therefore, in this study, we aimed to develop a clickable affinity-based probe (AfBP) to broaden the current possibilities to measure and detect the receptor.

**Figure 1:**
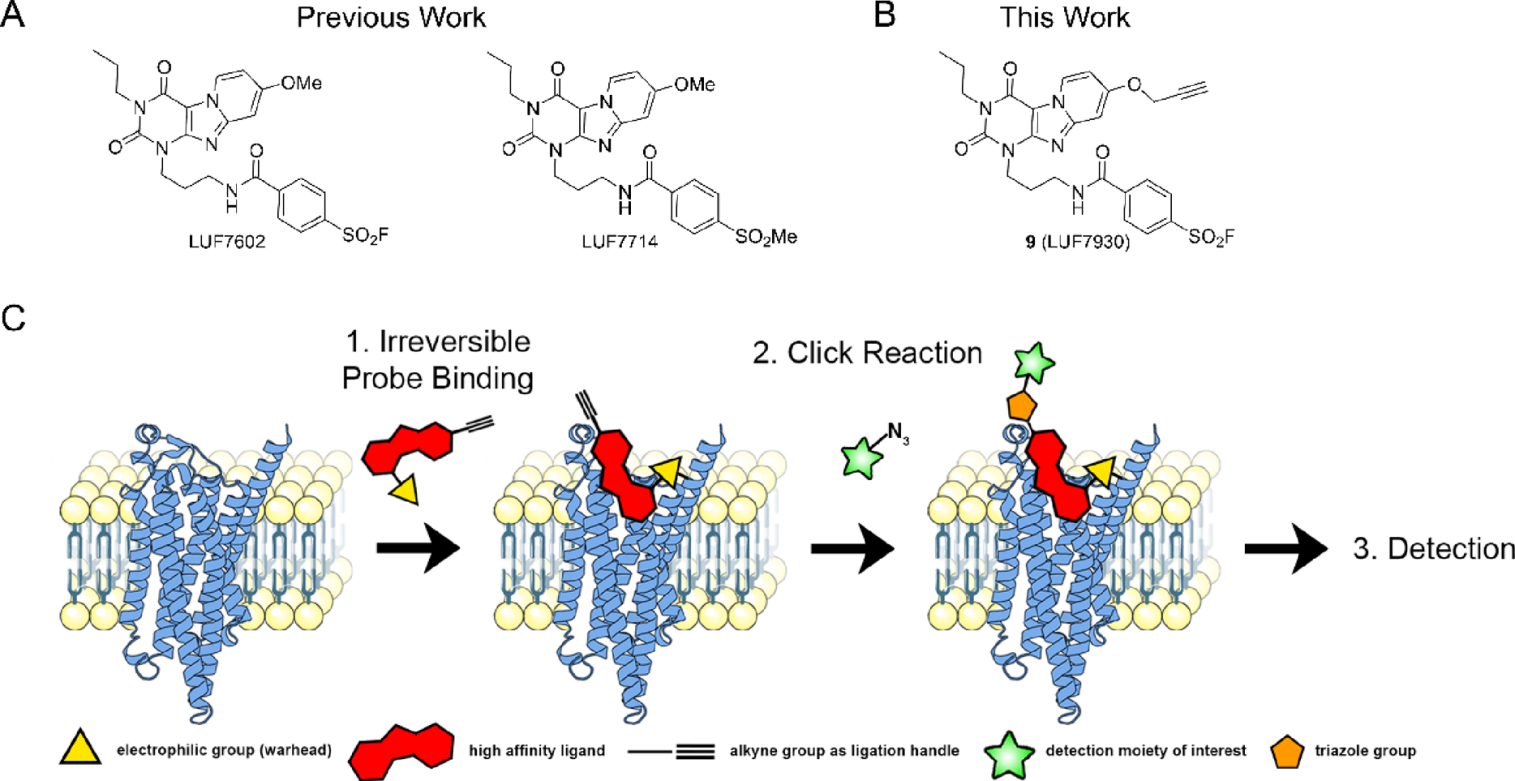
(A) Previous work: covalent hA_3_AR antagonist LUF7602 and control compound LUF7714; (B) this work: AfBP LUF7960; (C) strategy to label the hA_3_AR with an AfBP. First, the AfBP is added to cells or membrane fractions to allow irreversible bond formation between receptor and probe. Click reagents are then added to install a detection moiety onto the probe-bound receptor. Lastly, cells are further processed for detection, dependent on the detection method of interest. The image of the hA_3_AR was generated using Protein Imager,^29^ using the structure of the hA_3_AR (AF-P0DMS8-F1) as predicted by Alphafold.^30,31^

AfBPs are tool compounds that consist of three parts. First, an electrophilic group (‘warhead’) is incorporated, that facilitates covalent binding of the AfBP to the receptor (Figure 1).^32,33^ This allows usage of the probe in biochemical assays that rely on denaturation of proteins (e.g., SDS-PAGE and chemical proteomics). The warhead is coupled to a high affinity scaffold (the second part) that induces selectivity to the protein target of interest and thirdly, a detection moiety is introduced.

Our lab has recently reported the development of electrophilic antagonist LUF7602 as an irreversible ligand of the hA_3_AR (Figure 1A).^34^ LUF7602 contains two out of three functionalities of an AfBP, the only part missing being the detection moiety. In the past, trifunctional AR ligands have been synthesized containing both a warhead and a detection moiety.^35,36^ Here, we introduced an alkyne group as the ligation handle that can be ‘clicked’ to a detection moiety through the copper-catalyzed alkyne–azide cycloaddition (CuAAC).^37,38^ This approach resulted in a ‘modular’ probe that can be clicked in situ to any detection moiety of interest that contains an azide group. The new probe allowed specific detection of overexpressed hA_3_AR in various assay types, such as SDS-PAGE and confocal microscopy, as well as detection of endogenous hA_3_AR in flow cytometry experiments on granulocytes.

## Results and Discussion

### Design and Synthesis

**Scheme 1:**
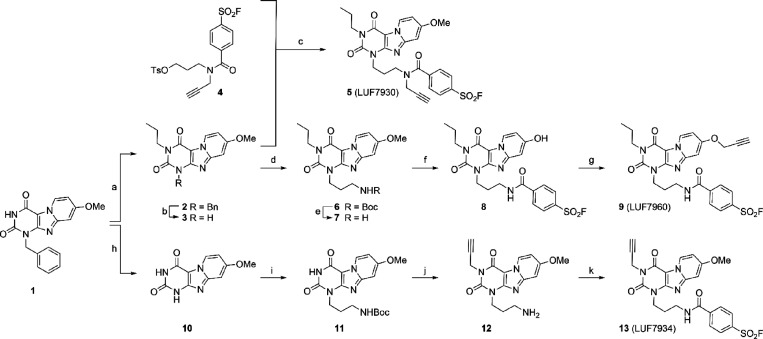
Synthesis of hA_3_AR-Targeting Affinity-Based Probes Reagents and conditions: (a) DBU, 1-bromopropane, MeCN, 70 °C, 1 h, quant.; (b) Pd(OH)_2_/C, NH_4_HCO_2_, EtOH, 80 °C, 7 days, 53%; (c) K_2_CO_3_, DMF, rt, overnight, 29%; (d) *tert*-butyl (3-bromopropyl)carbamate, K_2_CO_3_, DMF, 100 °C, 2 h, 97%; (e) TFA, CHCl_3_, 60 °C, overnight, 90%; (f) (i) BBr_3_ 1 M in DCM, CHCl_3_, 50 °C, 6 days; (ii) 4-fluorosulfonyl benzoic acid, EDC·HCl, DIPEA, DMF, rt, two days, 13% over two steps; (g) propargyl bromide (80% in toluene), K_2_CO_3_, DMF, rt, overnight, 30%; (h) Pd(OH)_2_/C, NH_4_HCO_2_, EtOH, 80 °C, 7 days; (i) *tert*-butyl (3-bromopropyl)carbamate, K_2_CO_3_, DMF, 0–40 °C, 6 days, 25%; (j) (i) propargyl bromide (80% in toluene), DBU, MeCN, rt, overnight; (ii) TFA, DCM, rt, 2 h, 67%; (k) 4-fluorosulfonyl benzoic acid, EDC·HCl, DIPEA, DMF, rt, 3 h, 13%.

We decided to incorporate an alkyne group into LUF7602 analogs, to enable click-based incorporation of a detection moiety of choice onto the AfBP. Similar click strategies have already been used in the synthesis of fluorescent ligands for the hA_3_AR.^20,21,26,27,39^ These ligands are however all lacking the electrophilic warhead. Additionally, contrary to those studies, we mainly performed the click reaction after binding of the AfBP to the receptor, preventing a loss of affinity due to bulky substituents. Such an approach has recently successfully been applied for the detection of multiple types of GPCR using photo-affinity probes^40−47^ as well as the detection of the A_1_AR and A_2A_AR using electrophilic probes.^48−50^ Also, a non-selective AfBP for the hA_3_AR has been reported, although detection of the hA_3_AR with this probe has yet to be validated.^48^ In previous studies on the A_1_AR, we observed that the position of the alkyne moiety on the scaffold can greatly influence the affinity of the AfBP toward the receptor, thereby affecting the functionality of the AfBP.^49^ To increase the chances of obtaining a successful AfBP, we therefore introduced the alkyne group on three divergent locations onto the scaffold of LUF7602 (Scheme 1).

All three synthetic routes started with compound **1**, a high-affinity selective antagonist for the hA_3_AR reported over two decades ago.^51^ First, **1** was alkylated with bromopropane yielding tricyclic compound **2**. The benzylic moiety was then removed using palladium over carbon and an excess of NH_4_HCO_2_.^34^ The secondary amine of **3** was alkylated with alkyne-containing fluorosulfonyl moiety **4**, synthesized as recently described,^49^ to yield compound **5** (LUF7930) as the first out of three AfBPs. For the second AfBP, the secondary amine of **3** was alkylated by a protected propylamine followed by deprotection of the Boc-group to yield compound **7**. The methoxy group of **7** was removed using BBr_3_, yielding a zwitterionic intermediate that, after removal of remaining BBr_3_, was used immediately in an amide bond formation to synthesize fluorosulfonyl derivative **8**. The alkyne moiety was then substituted onto the phenolic OH to yield compound **9** (LUF7960) as the second out of three AfBPs. For the last AfBP, the benzyl group of compound **1** was removed in the first step. However, synthesis and purification of **10** turned out to be cumbersome. Therefore, a crude mixture of **10** was used in the following alkylation step, resulting in a poor but sufficient yield of compound **11**. The alkyne moiety was then substituted onto compound **11** using propargyl bromide followed by deprotection of the Boc-group to yield compound **12**. Lastly, fluorosulfonyl benzoic acid was introduced using peptide coupling conditions, yielding compound **13** (LUF7934) as the final out of three AfBPs.

### Affinity and Selectivity toward the hA_3_AR

The affinity of the synthesized AfBPs was determined in radioligand binding experiments. First, single concentration displacement assays were carried out on all four human adenosine receptors, using a final probe concentration of 1 μM (Table 1). Over 90% displacement was observed on the hA_3_AR, while all probes showed minimal displacement (≤25%) of the radioligand on the other ARs. This quick screen indicated a good selectivity toward the hA_3_AR over the other human ARs, a trend that was also observed in the case of the parent compound.^34^ Next, the ‘apparent’ affinities (depicted as p*K*_i_ values) toward the hA_3_AR were determined using full curve displacement assays. To study the presumable covalent mode of action, the apparent affinity was determined with (pre-4 h) and without (pre-0 h) 4 h of pre-incubation of AfBP with hA_3_AR, prior to addition of radioligand. The three synthesized AfBPs showed very similar affinities at pre-0 h with apparent p*K*_i_ values in the double digit nanomolar range (Table 2). In all three cases, the apparent p*K*_i_ showed a strong increase upon 4 h of pre-incubation, toward values in the single-digit nanomolar range. This difference in affinity was reflected in a shift in apparent p*K*_i_. Hence, substitution of an alkyne moiety at all three of the divergent positions was well-tolerated in the case of binding affinity. To further investigate selectivity toward the hA_3_AR over the hA_1_AR, full curve displacement assays were carried out on the hA_1_AR. All AfBPs showed micromolar affinities at pre-0 h, with an increase toward submicromolar affinities at pre-4 h (Table S1). AfBP **5** showed the best selectivity toward the hA_3_AR over the hA_1_AR (>100-fold), while AfBPs **9** and **11** showed good selectivity (>17-fold and >7-fold, respectively).

**Table 1:** Radioligand Displacement (%) of the Synthesized hA_3_AR Probes on the Four Adenosine Receptors^e^

compound	hA_1_AR^a^	hA_2A_AR^b^	hA_2B_AR^c^	hA_3_AR^d^
**5** (LUF7930)	2	0	10	95
	(3, 0)	(0, 0)	(22, −2)	(96, 94)
**9** (LUF7960)	21	7	1	95
	(24, 18)	(11, 2)	(0, 2)	(94, 96)
**13** (LUF7934)	25	8	5	92
	(24, 26)	(9, 7)	(4, 5)	(90, 93)

a % specific [^3^H]DPCPX displacement by 1 μM of respective probe on CHO cell membranes stably expressing the human A_1_AR (hA_1_AR).

b % specific [^3^H]ZM241385 displacement by 1 μM of respective probe on HEK293 cell membranes stably expressing the human A_2A_AR (hA_2A_AR).

c % specific [^3^H]PSB-603 displacement by 1 μM of respective probe on CHO-spap cell membranes stably expressing the human A_2B_AR (hA_2B_AR).

d % specific [^3^H]PSB-11 displacement at 1 μM of respective probe on CHO cell membranes stably expressing hA_3_AR.

e Probes were co-incubated with radioligand for 30 min at 25 °C. Data represent the mean of two individual experiments performed in duplicate.

**Table 2:** Time-Dependent Apparent Affinity Values of the Synthesized hA_3_AR Probes^d^^e^

compound	p*K*_i_ (pre-0 h)^a^	p*K*_i_ (pre-4 h)^b^	fold change^c^
**5** (LUF7930)	7.55 ± 0.01	8.52 ± 0.05^d^	9.5 ± 1.0
**9** (LUF7960)	7.27 ± 0.07	8.40 ± 0.03^d^	13.5 ± 1.2
**13** (LUF7934)	7.17 ± 0.04	8.38 ± 0.05^d^	16.6 ± 3.5

a Apparent affinity determined from displacement of specific [^3^H]PSB-11 binding on CHO cell membranes stably expressing the hA_3_AR at 25 °C after 0.5 h of co-incubating the probe and radioligand.

b Apparent affinity determined from displacement of specific [^3^H]PSB-11 binding on CHO cell membranes stably expressing the hA_3_AR at 25 °C after 4 h of pre-incubation with the respective probe followed by an additional 0.5 h of co-incubation with the radioligand.

c Fold change determined by ratio *K*_i_(0 h)/*K*_i_(4 h).

d ****p < 0.0001 compared to the p*K*_i_ values obtained from the displacement assay with 0 h pre-incubation of the probe, determined by a one-way ANOVA test using multiple comparisons.

e Data represent the mean ± SEM of three individual experiments performed in duplicate.

**Figure 2:**
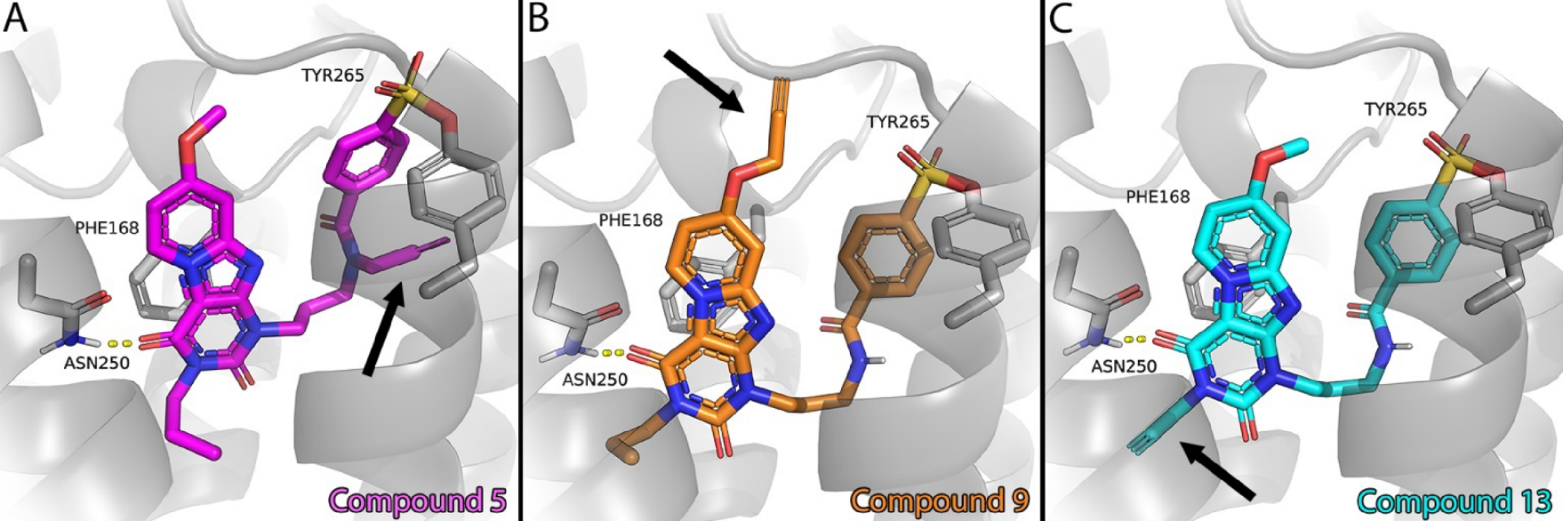
Putative binding modes of compounds **5** (A), **9** (B), and **13** (C) in an Alphafold model of the hA_3_AR (AF-P0DMS8-F1-model_v4).^30,31^ The extracellular side of the receptor is located at the top of the images, while the intracellular side is at the bottom. All three compounds show a hydrogen bond interaction with the conserved N250 and π–π stacking with Phe168, two well-known interactions in ligand recognition of adenosine receptors.^52^ The alkyne group, indicated with an arrow in each panel, fits into the binding pocket on each exit vector, and the binding orientation of the core compound, as published previously by our group, is maintained.^34^

**Figure 3:**
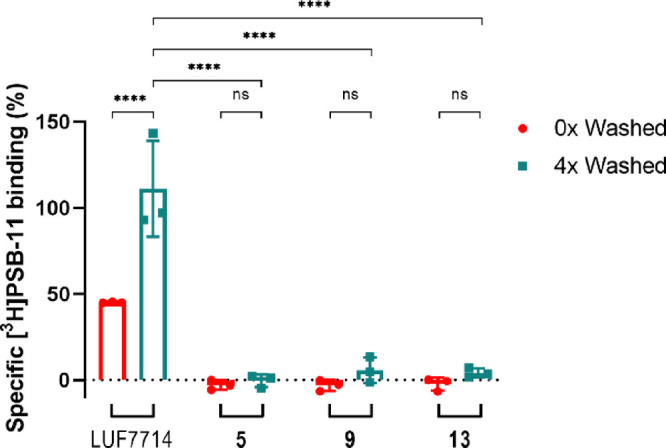
Wash-out assay reveals covalent binding of all three probes to the hA_3_AR. CHO cells membranes stably transfected with the hA_3_AR were pre-incubated with 1% DMSO (vehicle), 1 μM non-covalent control compound LUF7714, or 1 μM compounds **5**, **9**, or **13**. The samples were washed for either 0 or 4 times, before being exposed to [^3^H]PSB-11 in a radioligand displacement assay. Data is expressed as the percentage of the vehicle group (100%) and represents the mean ± SEM of three individual experiments performed in duplicate. ****p < 0.0001 determined by a two-way ANOVA test using multiple comparisons.

To investigate the binding mode of the probes within the orthosteric binding pocket, covalent docking experiments were performed using the previously determined nucleophile Y265 as the anchor (Figure 2).^34^ All three compounds show a hydrogen bond interaction with the conserved N250 and π–π stacking with Phe168, two well-known interactions in ligand recognition in adenosine receptors.^52^ Thus, the alkyne substitution seems to be well-tolerated for all three of the probes, thereby supporting the outcome of the radioligand displacement experiments. To further confirm the covalent mode of action, wash-out experiments were performed. Compounds **5**, **9**, and **13** all showed persistent binding to the hA_3_AR, while full recovery of radioligand binding was observed in the case of the reversible control compound LUF7714 (Figure 3, molecular structure in Figure 1A).^34^ Altogether, this indicates that the three synthesized AfBPs bind covalently to the hA_3_AR.

### Labeling of the hA_3_AR in SDS-PAGE experiments

**Figure 4:**
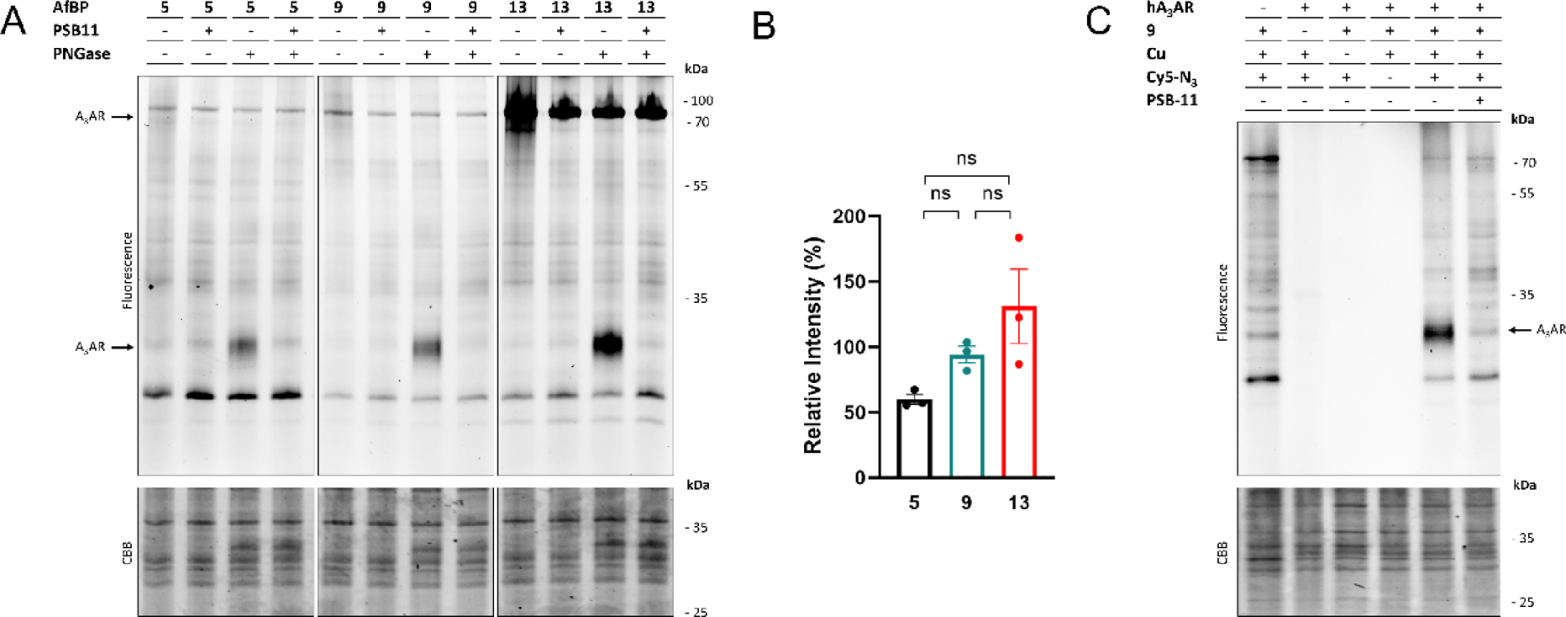
Labeling of the hA_3_AR by the synthesized affinity-based probes. (A) Labeling of proteins by **5**, **9**, and **13**. Membrane fractions from CHO cells stably overexpressing the hA_3_AR were pre-incubated for 30 min with the antagonist (PSB-11, 1 μM final concentration) or 1% DMSO (control), prior to incubation for 1 h with the respective probe (50 nM final concentration). The proteins were subjected to PNGase or MilliQ (control) for 1 h to remove N-glycans. Samples were then subjected to a copper-catalyzed click reaction with Cy5-N_3_ (1 μM final concentration), denatured using Laemmli buffer (4×) and resolved by SDS-PAGE. Gels were imaged using in-gel fluorescence and stained with Coomassie Brilliant Blue (CBB) as protein loading control. (B) Quantification of the lower hA_3_AR band (±30 kDa). The band intensities were taken and corrected for the observed amount of protein per lane upon CBB staining. The band at 55 kDa of the PageRuler Plus ladder (not shown) was set to 100% for each gel, and band intensities were calculated relative to this band. The mean values ± SEM of three individual experiments are shown. Significance was calculated using a one-way ANOVA test using multiple comparisons (ns = not significant). (C) Control experiments with probe **9**. Membrane fractions from CHO cells with or without (first lane) stable expression of the hA_3_AR were pre-incubated for 30 min with antagonist (PSB-11, 1 μM final concentration) or 1% DMSO (control), prior to incubation for 1 h with **9** (50 nM final concentration) or 1% DMSO (control). Proteins were deglycosylated with PNGase for 1 h. The click mix was then added, containing CuSO_4_ or MilliQ (control) and Cy5-N_3_ (1 μM final concentration) or DMSO (control). The samples were then denatured with Laemmli buffer (4×) and resolved by SDS-PAGE. Gels were imaged using in-gel fluorescence and afterward stained with CBB. The image shown is a representative of three individual experiments.

**Figure 5:**
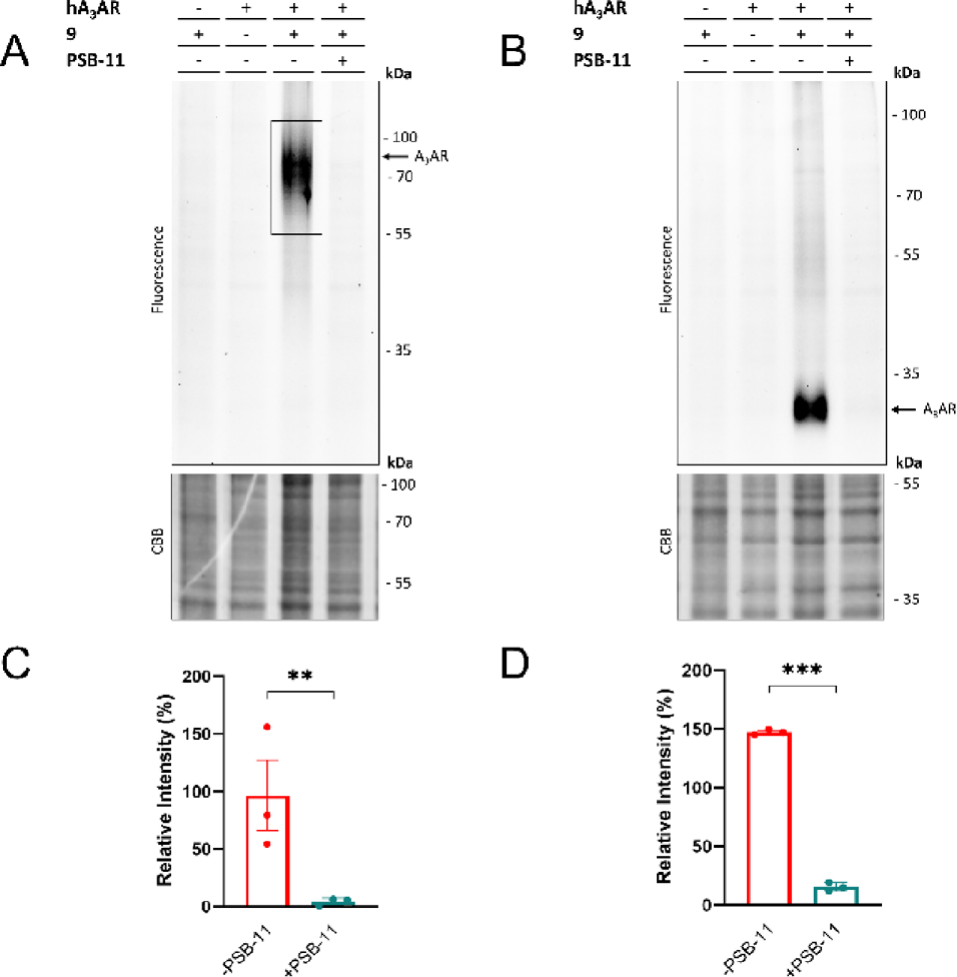
Labeling of the hA_3_AR on live CHO cells. CHO cells with or without (first lane) stable expression of the hA_3_AR were pre-incubated for 1 h with antagonist (PSB-11, 1 μM final concentration) at 37 °C, prior to incubation with **9** (50 nM final concentration) for 1 h at 37 °C. After the incubation, the unbound probe was washed away with PBS. Membranes were prepared, brought to a concentration of 1 μg/μL, and subjected to the copper-catalyzed click reaction with Cy5-N_3_ (1 μM final concentration). Samples were then denatured with Laemmli buffer (4×), resolved by SDS-PAGE, and imaged using in-gel fluorescence. Gels were stained by Coomassie Brilliant Blue (CBB) as loading control. (A) Labeling of glycosylated hA_3_AR. (B) Labeling of deglycosylated hA_3_AR. PNGase was added prior to the addition of click reagents. (C, D) Quantification of the observed signals with and without addition of antagonist (PSB-11). The band intensities were calculated using ImageLab and corrected for the amount of protein measured after CBB staining. The band at 55 kDa of the PageRuler Plus ladder (not shown) was set to 100% for each gel and band intensities were calculated relative to this band. The mean values ± SEM of three individual experiments are shown. Significance was calculated by a two-way ANOVA test using multiple comparisons (***p < 0.001; **p < 0.01).

Next, the potential AfBPs were investigated on their ability to label the hA_3_AR in SDS-PAGE experiments.^35,36^ Membrane fractions with stable expression of the hA_3_AR were incubated for 1 h with 50 nM (roughly the apparent *K*_i_) of AfBPs **5**, **9**, or **13**, subjected to a copper-catalyzed click ligation with sulfo-Cyanine5-azide (Cy5-N_3_), denatured, and resolved by SDS-PAGE. Pre-incubation with the hA_3_AR-selective antagonist PSB-11 was used as a negative control,^53^ and a subsequent incubation step with PNGase was introduced to remove N-glycans.^49^ Detection of labeled hA_3_AR turned out to be difficult if not deglycosylated: the receptor appeared as a smear at about 70 kDa (Figures 4A and 5A). Yet, this mass corresponds to the band of rat A_3_AR as has been shown in SDS-PAGE experiments on overexpressing CHO cells.^54,55^ N-Deglycosylation of the mixture of membrane proteins revealed a protein band at ±30 kDa in case of all three AfBPs. This protein was not labeled by any of the three AfBPs after pre-incubation with reversible antagonist PSB-11 and is therefore most likely the hA_3_AR. To select one of these AfBPs for further labeling studies, the intensities of the bands at ±30 kDa were compared between the probes (Figure 4B), but no significant differences were found. Correspondingly, the alkyne groups do not inflict any strong unfavorable interactions upon covalent docking of the compounds in the hA_3_AR model (Figure 2). Examination of the amount of off-target labeling indicated that there are fewer other proteins labeled by **9** than by the other two probes **5** and **13** (Figure 4A). Therefore, we decided to continue our subsequent experiments with AfBP **9**. Further control experiments were performed: labeling in CHO membrane fractions without expression of the hA_3_AR, no addition of probe and clicking without copper or Cy5-N_3_ (Figure 4C). The band at ±30 kDa was not observed in any of the control lanes, confirming that this band is the hA_3_AR. Notably, we did not observe any hA_3_AR-specific labeling by a commercially available hA_3_AR antibody in western blot experiments (Figure S1). Presumably, the selectivity and affinity of antibodies are compromised by the relatively short N-terminus and extracellular loops of the hA_3_AR.

### Labeling of hA_3_AR on Live CHO Cells

Having confirmed binding and labeling of the hA_3_AR in CHO membrane fractions, we moved toward labeling experiments on live CHO cells stably expressing the hA_3_AR. Live cells were incubated with 50 nM AfBP **9**, prior to processing for either SDS-PAGE or microscopy experiments. In the case of SDS-PAGE experiments, membranes were collected, and the probe-bound proteins were clicked to Cy5-N_3_, denatured, and resolved by SDS-PAGE. This yielded ‘cleaner’ gels as compared to labeling in membrane fractions: no strong off-target bands were observed (Figure 5). The smear of glycosylated hA_3_AR at ±70 kDa was more clearly visible as a single band (Figure 5A) and absent in the control lanes (no hA_3_AR, no AfBP or pre-incubation with PSB-11). Similar to the experiments on cell membranes, a strong reduction in size of the band was observed upon pre-incubation with PNGase (Figure 5B). Both signals were significantly reduced by pre-incubation with PSB-11 (Figure 5C,D). Thus, AfBP **9** also binds and labels the hA_3_AR on live cells. We speculate that the increased amount of off-target labeling in membrane fractions is due to the high enrichment of subcellular membrane proteins. Together with the electrophilic nature of the AfBP, this can result in an increased amount of protein labeling, as we have previously observed in our experiments with an electrophilic A_1_AR probe.^49^

**Figure 6:**
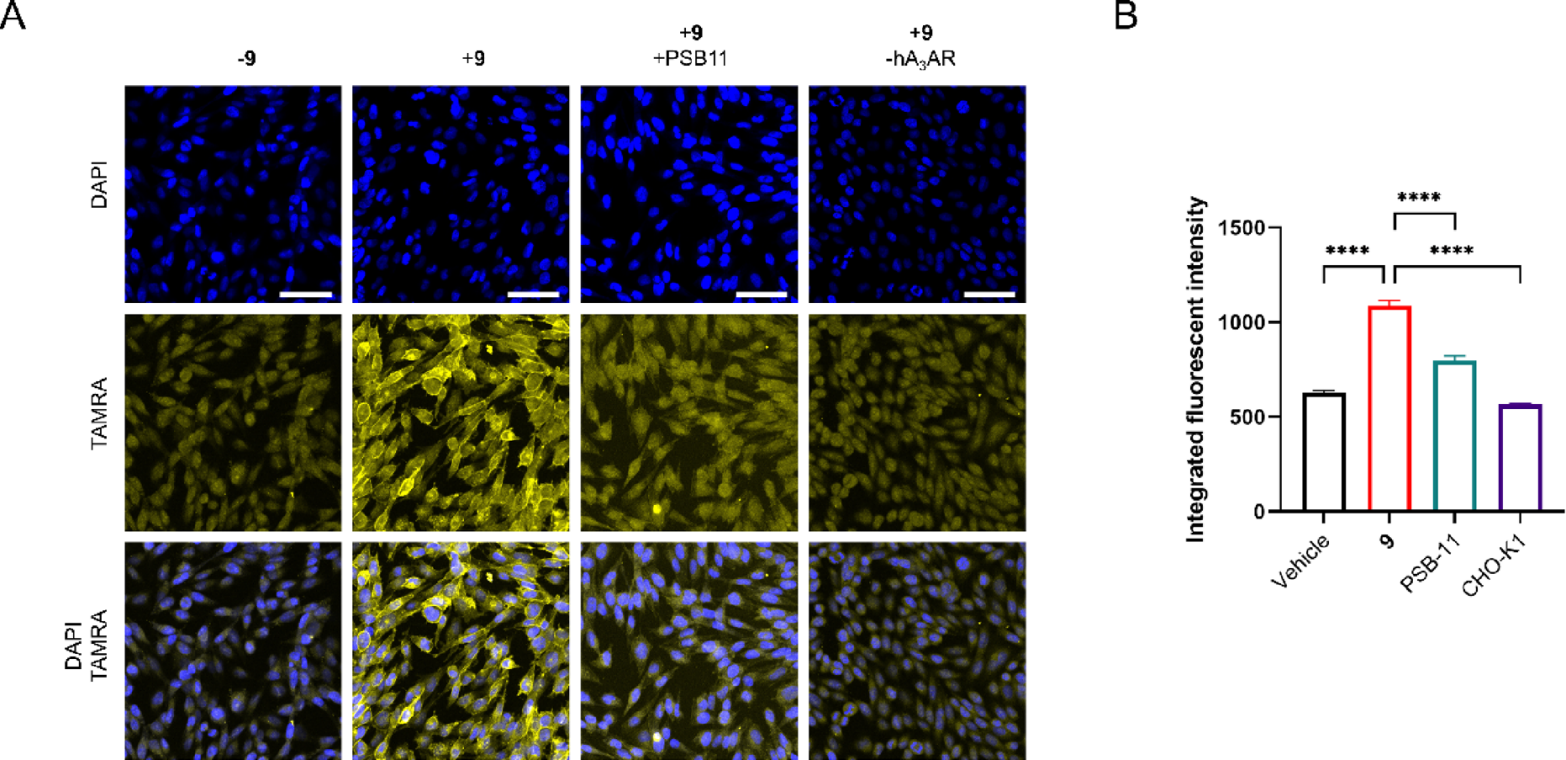
Labeling of the hA_3_AR observed by confocal microscopy. CHO cells with (CHO-hA_3_AR) or without (CHO-K1) stable expression of the hA_3_AR were pre-incubated for 30 min with PSB-11 (1 μM final concentration) or 1% DMSO (control) and incubated for 60 min with **9** or 1% DMSO (vehicle control). Cells were fixed, permeabilized, and subjected to a copper-catalyzed click reaction with TAMRA-N_3_ (1 μM final concentration). The cells were then washed and kept in PBS containing 300 nM DAPI during confocal imaging. (A) Shown are DAPI staining (blue, first row), TAMRA staining (yellow, second row), and an overlay of both stains (third row). Images were acquired automatically at multiple positions in the well of interest and are representatives from two biological experiments. Scale bar = 50 μM. Figure was created using OMERO.^57^ (B) Comparison of the integrated fluorescence intensity between treatment conditions. Data was obtained from 2 × 9 fields of view, from the same experiment performed in duplicate. Each data point represents the integrated fluorescent intensity of the TAMRA signal per individual cell. Shown in the bar graphs is the average integrated fluorescence intensity of all individual cells ± SEM. Significance was calculated using a one-way ANOVA test using multiple comparisons. A significant increase in intensity is observed for the cells containing the hA_3_AR and treated with **9**, versus the other conditions.

In the case of the microscopy experiments, cells were fixed after probe incubation followed by a click reaction with carboxytetramethylrhodamine azide (TAMRA-N_3_), chosen because of its uniform cellular distribution and diffusion.^56^ Next, multiple washing steps were carried out and cellular nuclei were stained with 4′,6-diamidino-2-phenylindole (DAPI) prior to confocal imaging. A strong increase in TAMRA intensity was observed at the cell membranes upon addition of the probe to the wells (Figure 6A), visible by the increase in signal at cell–cell contacts. This signal was reduced by pre-incubation with PSB-11 and was absent for CHO cells not expressing the hA_3_AR (Figure 6B). We therefore conclude that labeling of overexpressed hA_3_AR by **9** on living CHO cells can be studied by both SDS-PAGE and confocal microscopy experiments. Multiple fluorescent ligands have already been verified in similar fluorescence microscopy assays.^19,21−24,28^ Together these fluorescent ligands comprise a molecular ‘toolbox’ that allows extensive characterization of the hA_3_AR in microscopy assays, as well as localization and internalization (with fluorescent agonists) of the receptor.^12,24^ The introduction of an electrophilic warhead and clickable handle on probe **9** extends the possibilities to study the hA_3_AR in microscopy assays, e.g., by allowing workflows that are dependent on thorough washing steps and/or denaturation of proteins.

### Labeling of Endogenous hA_3_AR in Flow Cytometry Experiments

**Figure 7:**
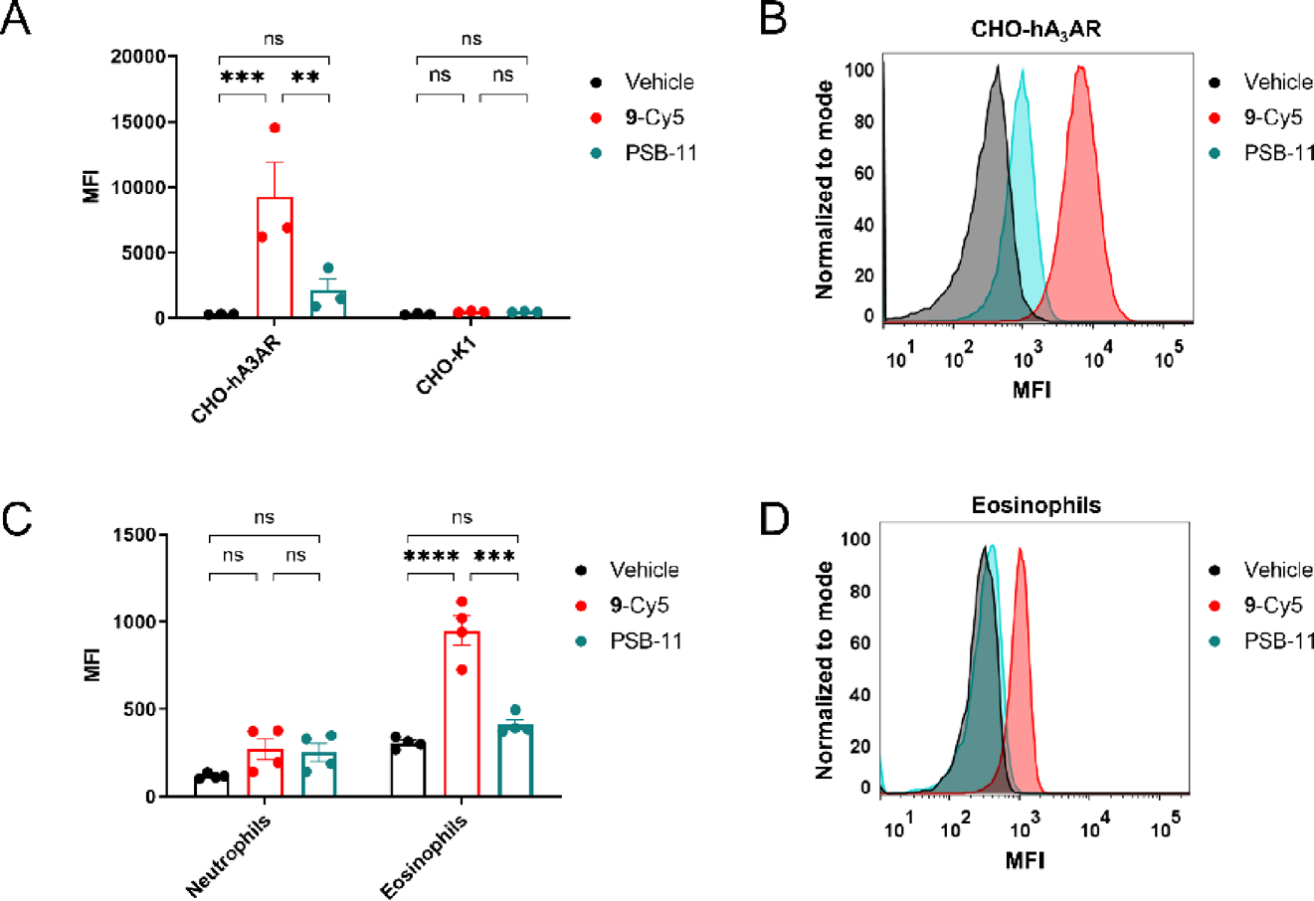
Labeling of the hA_3_AR in flow cytometry experiments. Samples were pre-incubated for 30 min with the antagonist PSB-11 and incubated for 60 min with **9** pre-clicked to a Cy5 fluorophore (**9**-Cy5). Samples were then washed and analyzed on Cy5 fluorescence by flow cytometry. (A) Cy5 mean fluorescence intensity (MFI) in CHO cells with (CHO-hA_3_AR) and without (CHO-K1) stable expression of the hA_3_AR. Values represent the mean ± SEM of three individual experiments performed in duplicate. Significance was calculated by a one-way ANOVA test using multiple comparisons (***p < 0.001; **p < 0.01; ns = not significant). (B) Representative graph showing the observed shift in MFI related to hA_3_AR labeling in CHO-hA_3_AR cells. (C) MFI of **9**-Cy5 in neutrophils and eosinophils purified from human blood samples. Values represent the mean ± SEM (*n* = 4) of four donors from two individual experiments. Significance was calculated by a one-way ANOVA test using multiple comparisons (****p < 0.0001; ***p < 0.001; ns = not significant). (D) Representative graph showing the observed shift in MFI related to hA_3_AR labeling in human eosinophils.

Having established the potential of **9** in overexpressing cell systems, we turned to native hA_3_AR expression in flow cytometry experiments in order to cope with expected low levels of observable fluorescence caused by the notoriously low expression levels of GPCRs. Similar usage of fluorescent probes in flow cytometry experiments have thus far led to kinetic studies of ligand binding,^20,27^ and detection of the hA_3_AR on the HL-60 model cell line.^21^ In order to establish an assay setup for primary cells we first used CHO cells as a model system. To avoid the use of excess copper on live cells, AfBP **9** was first clicked to Cy5-N_3_ and desalted, before further incubation steps. Pre-clicked **9**-Cy5 showed decrease in affinity toward the hA_3_AR (∼13-fold) and no binding toward the hA_1_AR in radioligand displacement assays (Table S2). **9**-Cy5 was then incubated for 1 h with living CHO cells with or without stable expression of the hA_3_AR. The unbound probe was removed by washing steps, and cells were analyzed by flow cytometry. The two types of CHO cells (+/– hA_3_AR) showed a difference in Cy5 mean fluorescence intensity (MFI), i.e., hA_3_AR-expressing CHO cells showed a significant increase in Cy5 MFI upon probe labeling, which was absent for CHO cells without hA_3_AR (Figure 7A,B). Next to that, pre-incubation with PSB-11 significantly reduced the observed signal in the case of the hA_3_AR-expressing CHO cells (Figure 7A,B), indicating that the observed signal is hA_3_AR-specific. Of note, no significant labeling was observed with a commercially available fluorophore-conjugated hA_3_AR antibody.

Next, we used the optimized procedure for further investigation of hA_3_AR expression on human granulocytes. Eosinophils and neutrophils were purified from human blood samples obtained from four donors, subjected to Cy5-clicked AfBP **9**, and analyzed by flow cytometry. Within these experiments, purified neutrophils did not show a significant increase in Cy5 MFI upon incubation with probe (Figure 7C). The purified eosinophils however showed a significant increase in MFI that was reduced upon pre-incubation with the antagonist PSB-11 (Figure 7C,D). Thus, with the aid of AfBP **9**, we were able to selectively detect hA_3_AR expression on human eosinophils, but not on human neutrophils. In the past, hA_3_AR expression has been observed on both human eosinophils and neutrophils,^7−9,12−14^ though the basal hA_3_AR expression levels on human neutrophils were low in comparison to the expression on stimulated neutrophils.^12,13^ Correspondingly, the herein investigated neutrophils showed little to no hA_3_AR expressed on the cell surface. Future studies that make use of AfBP **9** might therefore yield new information on hA_3_AR expression levels on stimulated neutrophils, among other leukocytes. Furthermore, the role of the hA_3_AR in inflammatory conditions, such as asthma, ischemic injury, and sepsis,^58−60^ might be elucidated using AfBP **9** as tool to detect receptor expression.

## Conclusions

In this work, we have synthesized and evaluated three affinity-based probes **5**, **9**, and **13**, which are all shown to bind covalently and with similar apparent affinities to the hA_3_AR. Although all three probes were able to label the hA_3_AR in SDS-PAGE experiments, we decided to continue with **9** due to the low number of off-target protein bands detected on gel. We showed that AfBP **9** is a versatile AfBP that can be used together with different fluorophores, examples in this study being Cy5 and TAMRA. The combination of **9** with click chemistry allowed us to label the hA_3_AR in various assay types, such as SDS-PAGE, confocal microscopy, and flow cytometry experiments, on both model cell lines and cells derived from human blood samples. In our hands, **9** showed to be successful in the selective labeling of the hA_3_AR, while commercial antibodies were not. We therefore believe that probe **9** will be of great use in the detection and characterization of the hA_3_AR in different types of granulocytes, among other cell types.

## Experimental Section

### General Chemistry

All reactions were carried out using commercially available reagents, unless noted otherwise. Reagents and solvents were purchased from Sigma-Aldrich (Merck), Fisher Scientific, or VWR chemicals. ZM241385 was kindly provided by Dr. S.M. Poucher (AstraZeneca), CGS21680 was purchased from Ascent Scientific, PSB 1115 potassium salt was purchased from Tocris Bioscience, and PSB-11 hydrochloride was purchased from Abcam Biochemicals. All reactions were carried out under a N_2_ atmosphere in oven-dried glassware. Reactions were monitored by thin layer chromatography (TLC) using TLC Silica gel 60 F254 plates (Merck) and UV irradiation (254 or 366 nM) as the detection method. ^1^H, ^13^C, and ^19^F NMR spectra were recorded on a Bruker AV-300 (300 MHz), Bruker AV-400 (400 MHz), or Bruker AV-500 (500 MHz) spectrometer. Chemical shift values are reported in ppm (δ) using tetramethylsilane (TMS) or solvent resonance as the internal standard. Coupling constants (*J*) are reported in Hz and multiplicities are indicated by s (singlet), d (doublet), dd (double doublet), t (triplet), q (quartet), p (pentuplet), h (hexuplet), or m (multiplet). Compound purity was determined by LC–MS, using the LCMS-2020 system (Shimadzu) coupled to a Gemini 3 μm C18 110 Å column (50 × 3 mm). In short, compounds were dissolved in H_2_O:acetonitrile (MeCN):*t*-BuOH 1:1:1, injected onto the column, and eluted with a linear gradient of H_2_O:MeCN 90:10 + 0.1% formic acid → H_2_O:MeCN 10:90 + 0.1% formic acid over the course of 15 min. High-resolution mass spectrometry (HRMS) was performed on a X500R QTOF mass spectrometer (SCIEX). All compounds are >95% pure by HPLC analysis.

#### 1-Benzyl-8-methoxypyrido[2,1-*f*]purine-2,4(1*H*,3*H*)-dione (**1**)

Compound **1** was synthesized in our lab as previously reported.^34^

#### 1-Benzyl-8-methoxy-3-propylpyrido[2,1-*f*]purine-2,4(1*H*,3*H*)-dione (**2**)

**1** (7.37 g, 22.9 mmol, 1.0 equiv) was dissolved in acetonitrile (145 mL), and DBU (9.58 mL, 64.1 mmol, 2.8 equiv) and 1-bromopropane (6.30 mL, 68.6 mmol, 3.0 equiv) were added. The mixture was stirred for 1 h at 70 °C and cooled with ice overnight. The solvent was then removed under vacuum. The residue was filtered and washed with H_2_O, EtOH, and Et_2_O and further dried under vacuum. This yielded **2** (quant., 8.33 g, 22.9 mmol) as a white solid. TLC (DCM:MeOH 98:2): R_f_ = 0.88. ^1^H NMR (400 MHz, (CD_3_)_2_SO): δ [ppm] = 8.81 (d, *J* = 7.4 Hz, 1H), 7.43–7.38 (m, 2H), 7.35 (t, *J* = 7.3 Hz, 2H), 7.33–7.26 (m, 2H), 6.98 (dd, *J* = 7.4, 2.6 Hz, 1H), 5.29 (s, 2H), 3.94 (s, 3H), 3.91 (t, *J* = 7.4 Hz, 2H), 1.63 (h, *J* = 7.5 Hz, 2H), 0.91 (t, *J* = 7.5 Hz, 3H). ^13^C NMR (101 MHz, (CD_3_)_2_SO): δ [ppm] = 162.2, 154.6, 151.8, 150.5, 137.7, 129.4, 128.7, 128.4, 128.3, 108.8, 101.0, 96.7, 57.2, 46.9, 42.9, 21.9, 12.1.

#### 8-Methoxy-3-propylpyrido[2,1-*f*]purine-2,4(1*H*,3*H*)-dione (**3**)

**2** (4.04 g, 11.1 mmol, 1.0 equiv), Pd(OH)_2_/C (4.05 g, 28.8 mmol, 2.6 equiv), and ammonium formate (0.72 g, 11.4 mmol, 1.0 equiv) were suspended in EtOH (500 mL) and stirred at 80 °C under reflux conditions. Over the course of 1 week, 3 extra portions of ammonium formate (0.72 g, 11.4 mmol, 1.0 equiv) were gradually added. The mixture was then cooled to rt and filtered over Celite. The residue was extracted with hot DMF, and the filtrate was concentrated. The residue was purified by column chromatography (DCM:MeOH 98:2 → 90:10) to yield **3** (1.60 g, 5.82 mmol, 53% yield) as a white solid. TLC (DCM:MeOH 95:5): R_f_ = 0.38. ^1^H NMR (400 MHz, (CD_3_)_2_SO): δ [ppm] = 12.05 (s, 1H), 8.74 (d, *J* = 7.4 Hz, 1H), 7.12 (d, *J* = 2.5 Hz, 1H), 6.90 (dd, *J* = 7.4, 2.5 Hz, 1H), 3.91 (s, 3H), 3.82 (t, *J* = 7.6 Hz, 2H), 1.63–1.52 (m, 2H), 0.88 (t, *J* = 7.3 Hz, 3H). ^13^C NMR (101 MHz, (CD_3_)_2_SO): δ [ppm] = 161.1, 154.5, 151.1, 150.5, 149.8, 127.5, 107.3, 95.4, 56.2, 41.0, 21.0, 11.2.

#### 3-(4-(Fluorosulfonyl)-*N*-(prop-2-yn-1-yl)benzamido)propyl-4-methylbenzenesulfonate (**4**)

Compound **4** was synthesized in our lab as previously reported.^49^

#### 4-((3-(8-Methoxy-2,4-dioxo-3-propyl-3,4-dihydropyrido[2,1-*f*]purin-1(2*H*)-yl)propyl)(prop-2-yn-1-yl)carbamoyl)benzenesulfonyl fluoride (**5**) (LUF7930)

To a solution of **3** (37 mg, 0.14 mmol, 1.0 equiv), **4** (63 mg, 0.14 mmol, 1.0 equiv) in dry DMF (1.5 mL) and K_2_CO_3_ (29 mg, 0.20 mmol, 1.5 equiv) were added. The reaction stirred over two nights at rt. The mixture was diluted with EtOAc (5 mL) and washed with water (2 × 5 mL). The water layers were combined and extracted with EtOAc (2 × 10 mL). The organic layers were combined, dried with MgSO_4_ and concentrated under reduced pressure. The crude product was purified with column chromatography (DCM:MeOH 98:2 → 95:5) to yield **5** (22 mg, 0.04 mmol, 29%) as a white solid. NMR measurements revealed the presence of two rotamers at 20 °C, but not at 100 °C. TLC (DCM:MeOH 98:2 + 1% Et_3_N): R_f_ = 0.63. ^1^H NMR (500 MHz, (CD_3_)_2_SO, 20 °C): δ [ppm] = 8.74 (dd, *J* = 23.1, 7.2 Hz, 1H), 8.25 (d, *J* = 8.3 Hz, 1H), 7.93 (d, *J* = 8.3 Hz, 1H), 7.81 (d, *J* = 8.6 Hz, 1H), 7.63 (d, *J* = 8.1 Hz, 1H), 7.23 (d, *J* = 15.1 Hz, 1H), 6.95 (t, *J* = 7.9 Hz, 1H), 4.35 (s, 1H), 4.16 (t, *J* = 6.2 Hz, 1H), 4.03 (s, 1H), 3.91 (d, *J* = 10.1 Hz, 5H), 3.78 (t, *J* = 6.8 Hz, 1H), 3.60 (t, *J* = 6.4 Hz, 1H), 3.40 (s, 1H), 3.32–3.24 (m, 1H), 2.19–2.12 (m, 1H), 2.11–2.03 (m, 1H), 1.64–1.56 (m, 1H), 1.56–1.47 (m, 1H), 0.86 (dt, *J* = 23.2, 7.4 Hz, 3H). ^1^H NMR (500 MHz, (CD_3_)_2_SO, 100 °C): δ [ppm] = 8.79 (d, *J* = 7.3 Hz, 1H), 8.09 (d, *J* = 7.8 Hz, 2H), 7.74 (d, *J* = 8.1 Hz, 2H), 7.15 (d, *J* = 2.7 Hz, 1H), 6.94 (dd, *J* = 7.4, 2.6 Hz, 1H), 4.20 (s, 2H), 4.10 (d, *J* = 5.3 Hz, 2H), 3.95 (s, 3H), 3.90 (t, *J* = 7.3 Hz, 2H), 3.52 (s, 2H), 3.11 (s, 1H), 2.16 (p, *J* = 6.6 Hz, 2H), 1.63 (h, *J* = 7.5 Hz, 2H), 0.90 (t, *J* = 7.5 Hz, 3H). ^13^C NMR (126 MHz, (CD_3_)_2_SO, 20 °C): δ [ppm] = 168.2, 167.9, 161.2, 153.7, 153.3, 150.9, 150.8, 150.5, 149.5, 149.4, 143.4, 132.4, 132.2, 132.0, 131.8, 128.9, 128.6, 128.3, 127.9, 127.7, 107.7, 100.1, 99.8, 95.6, 95.5, 79.3, 79.2, 75.9, 74.6, 56.2, 46.2, 42.8, 41.7, 40.7, 40.2, 38.8, 33.7, 26.9, 25.5, 20.9, 11.2. ^13^C NMR (126 MHz, (CD_3_)_2_SO, 100 °C) δ [ppm] = 167.7, 160.9, 153.2, 151.6, 150.5, 149.1, 143.0, 132.2 (d, *J* = 23.7 Hz), 127.9, 127.7, 127.2, 107.0, 99.6, 95.4, 78.6, 74.3, 55.7, 41.4, 40.1, 25.8, 20.3, 10.4. ^19^F NMR (471 MHz, (CD_3_)_2_SO, 20 °C): δ [ppm] = 67.08, 66.35. ^19^F NMR (471 MHz, (CD_3_)_2_SO, 100 °C): δ [ppm] = 65.99. HRMS (ESI, m/z): [M + H]^+^, calculated: 556.1661, found: 556.1628. HPLC 97%, RT 10.830 min.

#### *tert*-butyl-(3-(8-methoxy-2,4-dioxo-3-propyl-3,4-dihydropyrido[2,1-*f*]purin-1(2*H*)-yl)propyl)carbamate (**6**)

**3** (1.18 g, 4.3 mmol, 1.0 equiv), *tert*-butyl-(3-bromopropyl)carbamate (1.54 g, 6.5 mmol, 1.5 equiv), and K_2_CO_3_ (890 mg, 6.5 mmol, 1.5 equiv) were dissolved in DMF (60 mL) and refluxed at 100 °C for 2 h. Afterward, the mixture was allowed to cool down to rt overnight. The next day, the solvents were removed by evaporation under reduced pressure. The residue was dissolved in chloroform (100 mL), washed with H_2_O (3 × 100 mL), dried over MgSO_4_, and concentrated under reduced pressure to yield **6** (1.80 g, 4.2 mmol, 97%) as a white solid. TLC (DCM:MeOH 99:1): R_f_ = 0.76. ^1^H NMR (300 MHz, CDCl_3_): δ [ppm] = 8.82 (d, *J* = 7.3 Hz, 1H), 6.94 (d, *J* = 2.5 Hz, 1H), 6.75 (dd, *J* = 7.4, 2.5 Hz, 1H), 5.62 (s, 1H), 4.26 (t, *J* = 6.2 Hz, 2H), 4.01 (d, *J* = 7.5 Hz, 2H), 3.92 (s, 3H), 3.17–3.06 (m, 2H), 2.03–1.92 (m, 2H), 1.80–1.61 (m, 2H), 1.45 (s, 9H), 0.98 (t, *J* = 7.4 Hz, 3H). ^13^C NMR (75 MHz, CDCl_3_): δ [ppm] = 161.8, 156.2, 154.7, 151.9, 150.1, 128.1, 107.9, 95.3, 79.1, 56.0, 42.9, 40.9, 37.2, 28.6, 21.6, 11.5.

#### 1-(3-Aminopropyl)-8-methoxy-3-propylpyrido[2,1-*f*]purine-2,4(1*H*,3*H*)-dione (**7**)

TFA (12.8 mL, 166.6 mmol, 40.0 equiv) was added to a solution of **6** (1.80 g, 4.2 mmol, 1.0 equiv) in chloroform (40 mL), and the mixture was stirred at 60 °C overnight. Afterward, the pH was increased with 2 M NaOH (50 mL) to a value of approx. 10. The aqueous layer was extracted with EtOAc (2 × 50 mL) dried over MgSO_4_, filtered, and concentrated under reduced pressure to yield **7** (1.25 g, 3.8 mmol, 90%) as a white solid. TLC (DCM:MeOH 90:10 + 1% NEt_3_): R_f_ = 0.44. ^1^H NMR (400 MHz, CDCl_3_): δ [ppm] = 8.83 (dd, *J* = 7.3, 0.7 Hz, 1H), 6.95 (d, *J* = 1.9 Hz, 1H), 6.74 (dd, *J* = 7.4, 2.5 Hz, 1H), 4.29 (t, *J* = 6.7 Hz, 2H), 4.02 (t, *J* = 7.6 Hz, 2H), 3.92 (s, 3H), 2.74 (t, *J* = 6.5 Hz, 2H), 1.97 (p, *J* = 6.6 Hz, 2H), 1.71 (h, *J* = 7.4 Hz, 2H), 1.24 (t, *J* = 7.0 Hz, 2H), 0.98 (t, *J* = 7.4 Hz, 3H). ^13^C NMR (101 MHz, CDCl_3_): δ [ppm] = 161.6, 154.5, 151.5, 151.3, 149.9, 127.8, 107.6, 100.9, 95.1, 55.9, 42.6, 40.7, 38.8, 31.9, 21.4, 11.3.

#### 4-((3-(8-Hydroxy-2,4-dioxo-3-propyl-3,4-dihydropyrido[2,1-*f*]purin-1(2*d*)-yl)propyl)carbamoyl)benzenesulfonyl fluoride (**8**)

(i) BBr_3_ (1 M solution in DCM) (30.2 mL, 30.2 mmol, 10 equiv) was added to a solution of **7** (1.00 g, 3.0 mmol, 1.0 equiv) in CHCl_3_ (40 mL). The mixture was refluxed at 50 °C overnight. Additional BBr_3_ (1 M solution in DCM) (30.2 mL, 30.2 mmol, 10 equiv) was added, and the mixture was refluxed for 5 days. The reaction was then cooled to rt, upon which H_2_O (100 mL) was added dropwise to quench the reaction. The mixture was stirred for 1 h, and afterward the organic layer was removed. The aqueous layer was concentrated to yield the crude phenol. (ii) 4-Fluorosulfonyl benzoic acid (678 mg, 3.3 mmol, 1.1 equiv) and EDC·HCl (868 mg, 4.5 mmol, 1.5 equiv) were dissolved in dry DMF (10 mL) and stirred at rt. After 1 h, the mixture was added to a solution of the crude phenol (958 mg, 3.0 mmol, 1.0 equiv) in dry DMF (20 mL). DIPEA (1.8 mL, 10.6 mmol, 3.5 equiv) was added, and the mixture was stirred overnight. Additional 4-fluorosulfonyl benzoic acid (678 mg, 3.3 mmol, 1.1 equiv) and EDC·HCl (868 mg, 4.5 mmol, 1.5 equiv) were added. The mixture was stirred overnight, and the next day DCM (150 mL) was added. The organic layer was washed with 1 M HCl (2 × 200 mL), dried over MgSO_4_, filtered, and concentrated under reduced pressure. The residue was purified by column chromatography (DCM:MeOH 98:2 → 80:20) to yield **8** (194 mg, 0.4 mmol, 13% over two steps) as a white solid. TLC (DCM:MeOH 95:5): R_f_ = 0.45. ^1^H NMR (500 MHz, CDCl_3_): δ [ppm] = 8.77 (d, *J* = 7.1 Hz, 1H), 8.69 (t, *J* = 6.2 Hz, 1H), 8.18 (d, *J* = 8.1 Hz, 2H), 8.06 (d, *J* = 8.1 Hz, 2H), 7.96 (m, 1H), 6.96 (s, 1H), 6.80 (d, *J* = 7.0 Hz, 1H), 4.27 (t, *J* = 5.9 Hz, 2H), 3.99 (t, *J* = 7.4 Hz, 2H), 3.44 (m, 2H), 2.10 (m, 2H), 1.68 (h, *J* = 6.9 Hz, 2H), 0.93 (t, *J* = 7.4 Hz, 3H). ^13^C NMR (126 MHz, CDCl_3_): δ [ppm] = 165.5, 161.0, 154.3, 151.9, 151.3, 150.2, 141.0, 135.3 (d, *J* = 25.1 Hz), 128.8, 128.6, 108.3, 100.9, 98.1, 43.0, 40.9, 36.4, 27.4, 21.5, 11.4. ^19^F NMR (471 MHz, CDCl_3_): δ [ppm] = 65.58.

#### 4-((3-(2,4-Dioxo-8-(prop-2-yn-1-yloxy)-3-propyl-3,4-dihydropyrido[2,1-*f*]purin-1(2*H*)-yl)propyl)carbamoyl)benzenesulfonyl fluoride (**9**) (LUF7960)

A 80% v/v solution of propargyl bromide in toluene (0.42 mL, 0.4 mmol, 1.0 equiv) was further diluted in dry DMF (4.2 mL) and added to a solution of **8** (194 mg, 0.4 mmol, 1.0 equiv) in dry DMF (20 mL). K_2_CO_3_ (53 mg, 0.4 mmol, 1.0 equiv) was added to the mixture, which was stirred overnight. The mixture was then diluted with EtOAc (80 mL), and the organic layer was washed with H_2_O (2 × 100 mL) and brine (100 mL), dried over MgSO_4_, and concentrated under reduced pressure. The residue was purified by column chromatography (pentane:EtOAc 3:7) to yield **9** (63 mg, 0.1 mmol, 30%) as a white solid. TLC (EtOAc): R_f_ = 0.54. ^1^H NMR (500 MHz, CDCl_3_): δ [ppm] = 8.91 (d, *J* = 7.4 Hz, 1H), 8.47 (t, *J* = 6.3 Hz, 1H), 8.27 (d, *J* = 8.0 Hz, 2H), 8.18 (d, *J* = 8.5 Hz, 2H), 7.03 (d, *J* = 2.5 Hz, 1H), 6.85 (dd, *J* = 7.4, 2.5 Hz, 1H), 4.82 (d, *J* = 2.4 Hz, 2H), 4.35 (t, *J* = 5.8 Hz, 2H), 4.09–4.02 (m, 2H), 3.46 (q, *J* = 6.1 Hz, 2H), 2.64 (t, *J* = 2.4 Hz, 1H), 2.16 (p, *J* = 5.9 Hz, 2H), 1.74 (h, *J* = 7.5 Hz, 2H), 1.00 (t, *J* = 7.4 Hz, 3H). ^13^C NMR (126 MHz, CDCl_3_): δ [ppm] = 164.8, 159.8, 154.5, 152.1, 151.4, 149.5, 141.6, 135.4 (d, *J* = 29.1 Hz), 128.9, 128.6, 128.4, 108.3, 101.3, 96.6, 77.7, 76.6, 56.6, 43.0, 40.8, 35.8, 27.6, 21.6, 11.5. ^19^F NMR (471 MHz, CDCl_3_): δ [ppm] = 65.76. HRMS (ESI, *m*/*z*): [M + H]^+^, calculated: 542.1504, found: 542.1478. HPLC 98%, RT 10.875 min.

#### 8-Methoxypyrido[2,1-*f*]purine-2,4(1*H*,3*H*)-dione (**10**)

**1** (5.0 g, 15.51 mmol, 1.0 equiv) was suspended in EtOH (250 mL). Pd(OH)_2_/C (2.2 g, 15.5 mmol, 1.0 equiv) was added, and the reaction was brought under a nitrogen atmosphere. Ammonium formate (6.6 g, 105 mmol, 4.6 equiv) was added, and the mixture was refluxed at 80 °C. Over a period of 1 week, 10 portions of ammonium formate (6.6 g, 105 mmol, 4.6 equiv) were gradually added as well as one portion of extra Pd(OH)_2_/C (2.20 g, 15.5 mmol, 1.0 equiv). Afterward, the mixture was cooled to rt and filtered over Celite. The residue was extracted five times with hot DMF (100 mL). The filtrates were combined and concentrated. The residue was purified by column chromatography (DCM:MeOH 90:10 → 80:2) to yield 3.0 g of crude mixture **10**. This compound was used in subsequent reaction steps without further purifications. LC–MS [ESI + H]+: 233.00.

#### *tert*-Butyl-(3-(8-methoxy-2,4-dioxo-3,4-dihydropyrido[2,1-*f*]purin-1(2*H*)-yl)propyl)carbamate (**11**)

Crude **10** (1.57 g, 6.8 mmol, 1.0 equiv) was dissolved in dry DMF (70 mL), and *tert*-butyl-(3-bromopropyl)carbamate (1.61 g, 6.8 mmol, 1.0 equiv) was added. The mixture was cooled to 0 °C, and K_2_CO_3_ was added (1.40 g, 10.1 mmol, 1.5 equiv). The mixture was allowed to warm up to rt overnight and was stirred for another 3 days. Due to the slow progress of the reaction, the mixture was heated at 40 °C and stirred for another 2 days. Extra *tert*-butyl-(3-bromopropyl)carbamate was then added (0.32 g, 1.4 mmol, 0.2 equiv), which resulted in the slow formation of double substituted **3** (as determined by LC–MS). The reaction was therefore stopped. EtOAc (180 mL) was added, and the organic layer was washed with H_2_O (3 × 180 mL) and brine (90 mL), dried over MgSO_4_, filtered, and concentrated under reduced pressure. The residue was purified by column chromatography (DCM:MeOH 98:2 → 95:5) to yield **11** (486 mg, 1.68 mmol, 25%). LC–MS [ESI + H]+: 390.15.

#### 1-(3-Aminopropyl)-8-methobxy-3-(prop-2-yn-1-yl)pyrido[2,1-*f*]purine-2,4(1*H*,3*H*)-dione (**12**)

(i) Propargylbromide (80% in toluene) (446 μL, 4.14 mmol, 3.0 equiv) and DBU (0.619 mL, 4.14 mmol, 3.0 equiv) were added to a mixture of **11** (536 mg, 1.38 mmol, 1.0 equiv) in acetonitrile (7 mL), upon which the suspension became a clear solution. The mixture was stirred overnight and the next day H_2_O (25 mL) was added. The aqueous layer was extracted with DCM (2 × 35 mL). The organic layers were combined, dried over MgSO_4_, filtered, and concentrated under reduced pressure. (ii) The resulting residue was dissolved in DCM (10 mL), and TFA (5 mL) was added. The mixture was stirred for 2 h and afterward quenched by the addition of 2 M NaOH (50 mL). The aqueous layer was extracted with EtOAc (2 × 50 mL). The organic layers were combined, dried with MgSO_4_, filtered, and concentrated under reduced pressure. The residue was purified by column chromatography (DCM:MeOH 95:5 → 85:15) to yield **12** (300 mg, 0.92 mmol, 67%) as an off-white solid. TLC (DCM:MeOH 85:15 + 1% Et_3_N): R_f_ = 0.67. ^1^H NMR (500 MHz, CD_3_OD): δ [ppm] = 8.69 (d, *J* = 7.3 Hz, 1H), 6.99 (d, *J* = 2.5 Hz, 1H), 6.83 (dd, *J* = 7.4, 2.5 Hz, 1H), 4.70 (d, *J* = 2.4 Hz, 2H), 4.23 (t, *J* = 6.4 Hz, 2H), 3.90 (s, 3H), 2.99 (t, *J* = 7.2 Hz, 2H), 2.48 (t, *J* = 2.4 Hz, 1H), 2.15 (p, *J* = 6.8 Hz, 2H).

#### 4-((3-(8-Methoxy-2,4-dioxo-3-(prop-2-yn-1-yl)-3,4-dihydropyrido[2,1-*f*]purin-1(2*H*)-yl)propyl)carbamoyl)benzenesulfonyl fluoride (**13**) (LUF7934)

EDC·HCl (347 mg, 1.81 mmol, 2.0 equiv), 4-(fluorosulfonyl)benzoic acid (347 mg, 1.70 mmol, 1.9 equiv), and DIPEA (387 μL, 2,26 mmol) were added to a solution of **12** (300 mg, 0.92 mmol, 1.0 equiv) in dry DMF (6 mL). The mixture was stirred for 3 h, after which all starting materials were consumed (as determined by TLC). DCM (50 mL) was added, and the organic layer was washed with water (50 mL) and brine (50 mL), dried over MgSO_4_, filtered, and concentrated under reduced pressure. The residue was purified by column chromatography (DCM:MeOH 99:1 → 98:2) and (pentane:EtOAc 1:1 → 0:1) to yield **13** (60 mg, 0.12 mmol, 13%) as a white solid. TLC (DCM:MeOH 99:1): R_f_ = 0.35. ^1^H NMR (500 MHz, CDCl_3_): δ [ppm] = 8.85 (d, *J* = 7.3 Hz, 1H), 8.27 (t, *J* = 6.2 Hz, 1H), 8.24 (d, *J* = 8.3 Hz, 2H), 8.14 (d, *J* = 8.5 Hz, 2H), 6.86 (d, *J* = 2.5 Hz, 1H), 6.82 (dd, *J* = 7.3, 2.5 Hz, 1H), 4.86 (d, *J* = 2.4 Hz, 2H), 4.37 (t, *J* = 6.0 Hz, 2H), 3.92 (s, 3H), 3.48 (q, *J* = 6.0 Hz, 2H), 2.21 (t, *J* = 2.4 Hz, 1H), 2.20–2.14 (m, 2H). ^13^C NMR (126 MHz, CDCl_3_): δ [ppm] = 165.0, 162.5, 153.3, 151.5, 151.2, 149.9, 141.6, 135.4 (d, *J* = 25.4 Hz), 128.8, 128.7, 128.4, 108.6, 101.0, 95.1, 78.5, 71.0, 56.2, 41.2, 36.0, 30.6, 27.5. ^19^F NMR (471 MHz, CDCl_3_): δ [ppm] = 65.71. HRMS (ESI, *m*/*z*): [M + H]^+^, calculated: 514.1191, found: 514.1149. HPLC 100%, RT 10.077 min.

### Docking of the Affinity-Based Probes in an hA_3_AR Homology Model

The Alphafold model of the hA_3_AR was retrieved from the GPCRdb (AF-P0DMS8-F1-model_v4).^30,31,61^ This structure was prepared using the protein preparation wizard in Maestro (version 13.3, 2022–3, Schrödinger, LLC),^62^ and the four compounds (compound **5**, **9**, and **13** from this work and **17b** (LUF7602) from Yang et al.)^34^ were prepared using LigPrep (Schrödinger, LLC).^63^ Compound **17b** (LUF7602) was docked in the receptor model using the covalent binding protocol^64^ and consecutively energy-minimized using MacroModel (Schrödinger, LLC). The alkyne moiety was introduced on each of the exit vectors in 3D and subsequently subjected to an energy minimization step using MacroModel. Each of the resulting energy minimized structures was visualized using PyMOL (The PyMOL Molecular Graphics System, version 1.2r3pre, Schrödinger, LLC).

### Ethics Approval

The parts of the study involving human participants were reviewed and approved by the Sanquin Institutional Ethical Committee (project number NVT0606.01). All blood samples were obtained after informed consent and according to the Declaration of Helsinki 1964. The patients and participants provided informed consent to participate in this study.

### Cell Lines

Chinese hamster ovary (CHO) cells stably expressing the human adenosine A_3_ receptor (CHOhA_3_AR) were kindly provided by Prof. K.N. Klotz (University of Würzburg). CHO cells stably expressing the human adenosine A_1_ receptor (CHOhA_1_AR) were kindly provided by Prof. S.J. Hill (University of Nottingham), human embryonic kidney 293 (HEK293) cells stably expressing the human adenosine A_2A_ receptor (HEKhA_2A_AR) were kindly provided by Dr. J. Wang (Biogen), and CHO cells stably expressing the human A_2B_ receptor (CHO-spap-hA_2B_AR) were kindly provided by S.J. Dowell (GlaxoSmithKline).

### Cell Culture and Membrane Preparation

CHOhA_3_AR, CHOhA_1_AR, CHOhA_2A_AR, CHO-spap-hA_2B_AR, and CHO-K1 cells were cultured as previously reported.^65^ Membranes were prepared in the following manner: cells were detached from plates by scraping in PBS (5 mL). The cells were collected and centrifuged (5 min, 1000 rpm). The supernatant was removed, and cells were resuspended in ice-cold Tris–HCl buffer (pH 7.4). The cells were homogenized (Heidolph Diax 900 homogenizer), and the membranes were separated from the cytosolic fraction by centrifugation (20 min, 31,000 rpm, 4 °C) using a Beckman Optima LE-80K ultracentrifuge. The pellet was resuspended in Tris–HCl buffer, and the homogenization and centrifugation steps were repeated. The resulting pellet was resuspended in Tris–HCl buffer, and ADA was added (0.8 U/mL) to break down endogenous adenosine. Total protein concentrations were determined using the BCA method.^66^

### Purification of Human Granulocytes

Primary cells were isolated from human blood collected from healthy donors. Polymorphonuclear neutrophils (PMNs) were isolated using a Percoll gradient with a density of 1.076 g/mL.^67^ Erythrocytes were lysed with isotonic NH_4_Cl/KHCO_3_, washed twice in PBS, and resuspended in HEPES buffer (20 mM HEPES, 132 mM NaCl, 6.0 mM KCl, 1.0 mM CaCl_2_, 1.0 mM MgSO_4_, 1.2 mM KH_2_PO_4_, 5.5 mM glucose, and 0.5% (w/v) human serum albumin, pH 7.4). Eosinophils were isolated from the PMNs as described before.^68^

### Biologicals

PNGase (cat# V4831) was purchased from Promega (Leiden, The Netherlands). RabbitαhA_3_AR antibody (cat# bs-1225R) was ordered from Thermo Scientific (Landsmeer, The Netherlands), rabbitαhA_3_AR PE conjugate (cat# orb495084) was ordered from Biorbyt (Huissen, The Netherlands), goatαrabbit-HRP antibody (cat# 115–035-003) was purchased from Brunschwig Chemie (Amsterdam, The Netherlands), and PE anti-human Siglec-8 Antibody (mouse IgG1 clone 7C9) (cat# 347104) was purchased from Biolegend (San Diego, CA, USA). Bovine Serum Albumin (BSA)(cat# 268131000) was purchased from Acros Organics (Geel, Belgium).

### Radioligands

[^3^H]PSB-11, specific activity 56 Ci/mmol, was a kind gift from Prof. C.E. Müller (University of Bonn). [^3^H]DPCPX, specific activity 137 Ci/mmol, and [^3^H]ZM241385, specific activity 50 Ci/mmol, were purchased from ARC Inc., and [^3^H]PSB-603, specific activity 79 Ci/mmol, was purchased from Quotient Bioresearch.

### Single-Point Radioligand Displacement Assay on All Four Adenosine Receptors

Membrane aliquots containing 15 μg (CHOhA_3_AR), 5 μg (CHOhA_1_AR), or 30 μg (HEK293hA_2A_AR and CHO-spap-A_2B_AR) of protein were resuspended in assay buffer (A_3_AR: 50 mM Tris–HCl, pH 8.0, 10 mM MgCl_2_, 1 mM EDTA, 0.01% CHAPS; A_1_AR and A_2A_AR: 50 mM Tris–HCl, pH 7.4; A_2B_AR: 50 mM Tris–HCl, pH 7.4, 0.1% CHAPS). The competing ligand (1 μM) and radioligand (A_3_AR: 10 nM [^3^H]PSB-11; A_1_AR: 1.6 nM [^3^H]DPCPX; A_2A_AR: 1.7 nM [^3^H]ZM241385; A_2B_AR: 1.5 nM [^3^H]PSB-603) were added, and the samples were incubated in a total volume of 100 μL of the respective assay buffer for 30 min at 25 °C. Nonspecific binding was determined in the presence of 100 μM NECA (A_3_AR), 100 μM CPA (A_1_AR), 100 μM NECA (A_2A_AR), and 10 μM ZM241385 (A_2B_AR). Incubations were terminated by rapid vacuum filtration to separate the bound and free radioligand through prewetted 96-well GF/C filter plates using a PerkinElmer Filtermate harvester. Filters were subsequently washed 12 times with ice-cold wash buffer (A_3_AR: 50 mM Tris–HCl, pH 8.0, 10 mM MgCl_2_, 1 mM EDTA; A_1_AR and A_2A_AR: 50 mM Tris–HCl, pH 7.4; A_2B_AR: 50 mM Tris–HCl, pH 7.4, 0.1% BSA). The plates were dried at 55 °C, and MicroscintTM-20 cocktail (PerkinElmer) was added subsequently. After 3 h, the filter-bound radioactivity was determined by scintillation spectrometry using a 2450 MicroBeta Microplate Counter (PerkinElmer).

### Full Curve Radioligand Displacement Assay on the Adenosine A_3_ Receptor

Membrane aliquots containing 15 μg of protein (CHOhA_3_AR) were resuspended in assay buffer (50 mM Tris–HCl, pH 8.0, 10 mM MgCl_2_, 1 mM EDTA, 0.01% CHAPS). The competing ligand (concentrations ranging from 0.1 to 1000 nM) was added and pre-incubated with the membrane fractions for either 0 or 4 h. The radioligand (10 nM [^3^H]PSB-11) was then added and incubated with the samples in a total volume of 100 μL of assay buffer for 30 min at 25 °C. Nonspecific binding was determined in the presence of 100 μM NECA. Incubations were terminated by rapid vacuum filtration through prewetted 96-well GF/C filter plates using a PerkinElmer Filtermate harvester. Filters were subsequently washed 12 times with ice-cold wash buffer (50 mM Tris–HCl, pH 8.0, 10 mM MgCl_2_, 1 mM EDTA). The plates were dried at 55 °C, and MicroscintTM-20 cocktail (PerkinElmer) was added subsequently. After 3 h, the filter-bound radioactivity was determined using a Tri-Carb 2810TR Liquid Scintillation Analyzer (PerkinElmer).

### Wash-out Assay

200 μL assay buffer (50 mM Tris–HCl, pH 8.0, 10 mM MgCl_2_, 1 mM EDTA and 0.01% CHAPS), 100 μL assay buffer containing the competing ligand (final concentration: 1 μM), and 100 μL of CHOhA_3_AR membrane fractions (100 μg protein) were combined and pre-incubated for 2 h at 25 °C while shaking. The ‘4× washed’ samples were centrifuged (5 min, 13,000 rpm, 4 °C), and the supernatant was removed. The resulting pellet was dissolved in 1 mL of assay buffer and incubated for 10 min at 25 °C while shaking. The ‘4× washed’ samples were then again centrifuged (2 min, 13,000 rpm, 4 °C), the supernatant was removed, and the resulting pellet was dissolved in 1 mL of assay buffer. The latter washing steps were repeated two times (four times washing total), and the final pellet was dissolved in 300 μL assay buffer. The radioligand (10 nM [^3^H]PSB-11) in 100 μL of assay buffer was added to all samples, and the samples were incubated at 25 °C for 1 h. Nonspecific binding was determined in the presence of 100 μM NECA. Incubations were terminated by addition of 1 mL of ice-cold washing buffer (50 mM Tris–HCl, pH 8.0, 10 mM MgCl_2_, 1 mM EDTA) and rapid vacuum filtration through prewetted 96-well GF/B filter plates using a Brandel harvester. Filters were subsequently washed 5 times with ice-cold wash buffer. The plates were dried under vacuum, and a MicroscintTM-20 cocktail (PerkinElmer) was added subsequently. After 3 h, the filter-bound radioactivity was determined using a 2450 MicroBeta Microplate Counter (PerkinElmer).

### Data Analysis of Radioligand Displacement Assays

Data analysis was performed using GraphPad Prism version 9.0.0 (San Diego, California USA). pIC_50_ values were obtained by non-linear regression curve fitting and converted to p*K*_i_ values using the Cheng–Prusoff equation.^69^ As such, the K_D_ values of 1.6 nM of [^3^H]DPCPX at CHOhA_1_AR membranes and 1.7 nM of [^3^H]PSB603 at CHO-spap-hA_2B_AR membranes were taken from previous experiments,^70,71^ while the K_D_ values of 1.0 nM of [^3^H]ZM241385 at HEK293hA_2A_AR membranes and 17.3 nM of [^3^H]PSB-11 at CHOhA_3_AR membranes were taken from in-house determinations. Single point displacement values shown are mean percentages of two individual experiments performed in duplicate. All p*K*_i_ values shown are mean values ± SEM of three individual experiments performed in duplicate. Statistical analysis was performed using a one-way ANOVA test with multiple comparisons (****p < 0.0001).

### SDS-PAGE Experiments Using Membranes

Membrane fractions were prepared as mentioned above and diluted to a concentration of 1 μg/μL. 18 μL of membrane fractions from either CHOhA_3_AR or CHO-K1 cells were taken. 1 μL of competing ligand (PSB-11, unless noted otherwise) was added (final concentration: 1 μM in 0.05% DMSO in assay buffer), and the samples were shaken for 30 min at rt. 1 μL of affinity-based probe was then added (final concentration: 50 nM in 0.05% DMSO in assay buffer, unless noted otherwise), and the samples were shaken for 1 h at rt. Deglycosylation was initiated by addition of 0.5 μL of PNGaseF (5 U), 0.5 μL of MilliQ water for the control samples, and the samples were shaken for 1 h at rt. The click mix was freshly prepared by adding together 5 parts 100 mM CuSO_4_ in MilliQ, 3 parts 1 M sodium ascorbate (NaAsc) in MilliQ, 1 part 100 mM Tris(3-hydroxypropyltriazolylmethyl)amine (THPTA) in MilliQ, and 1 part 100 μM Cy5-N_3_ in DMSO. 2.28 μL of click mix was added to the samples (final concentration Cy5-N_3_: 1 μM in 1% DMSO in MilliQ), and the samples were shaken for 1 h at rt. Lastly, 7.59 μL of 4× Laemmli buffer was added and the samples were shaken for at least 1 h at rt. The samples were then loaded on gel (12.5% acrylamide) and run (180 V, 100 min). In-gel fluorescence was measured on a Bio-Rad Universal Hood III using Cy3 (605/50 filter) or Cy5 (695/55 filter) settings. A Pageruler prestained protein ladder was used as the molecular weight marker. After scanning, gels were either transferred to 0.2 μM PVDF blots (Bio-Rad) using a Bio-Rad Trans-Blot Turbo system (2.5 A, 7 min) or stained with Coomassie Brilliant Blue.

### SDS-PAGE Experiments Using Live CHO Cells

CHOhA_3_AR and CHO-K1 cells were cultured as mentioned above. Cells were grown to ∼90% confluency in 10 cm φ plates. 10 plates were used per condition per experiment. The medium was removed, and the competing ligand (PSB-11, unless noted otherwise) in medium (final concentration: 1 μM) was added. Cells were incubated for 1 h (37 °C, 5% CO_2_). The medium was removed, and the affinity-based probe (final concentration: 50 nM in medium) was added. The cells were incubated for 1 h (37 °C, 5% CO_2_) and washed with PBS to get rid of all the non-bound probes. Subsequently, the membranes were prepared and collected using the procedure as described above. Membrane aliquots were diluted to a concentration of 1 μg/μL, and 20 μL was taken per sample. PNGase (0.5 μL, 5 U) or MilliQ was added, and the samples were incubated for 1 h at rt. Next, the click mix was freshly prepared by adding together 5 parts 100 mM CuSO_4_ in MilliQ, 3 parts 1 M NaAsc in MilliQ, 1 part 100 mM THPTA in MilliQ, and 1 part 100 μM Cy5-N_3_ in DMSO. 2.28 μL of click mix was added per sample (final concentration Cy5-N_3_: 1 μM in 1% DMSO in MilliQ), and the samples were shaken for 1 h at rt. 7.59 μL of 4× Laemmli buffer was added per sample, and the samples were shaken for at least 1 h at rt. The samples were then loaded on gel (12.5% acrylamide) and run (180 V, 100 min). In-gel fluorescence was measured on a Bio-Rad Universal Hood III using Cy3 (605/50 filter) or Cy5 (695/55 filter) settings. A Pageruler prestained protein ladder was used as the molecular weight marker. After scanning, gels were either transferred to 0.2 μM PVDF blots (Bio-Rad) using a Bio-Rad Trans-Blot Turbo system (2.5 A, 7 min) or stained with Coomassie Brilliant Blue.

### Western Blot Experiments

Blots were blocked for 1 h at rt in 5% BSA in TBST. Primary antibody RabbitαhA_3_AR (bs-1225R) 1:10.000 in 1% BSA in TBST was then added, and the blots were incubated overnight at 4 °C. Blots were washed (3 × TBST) and incubated for 1 h at rt with secondary antibody goatαrabbit-HRP (115-035-003) 1:2.000 in 1% BSA in TBST. The blots were washed (2 × TBST and 1 × TBS) and activated by incubating 3 min in the dark using 1 mL of both reagents of Pierce ECL Western Blotting Substrate. Blots were scanned on chemiluminescence and fluorescence using the Bio-Rad Universal Hood III.

### Data Analysis of SDS-PAGE Experiments

Gel images were analyzed using Image Lab software version 6.0.1 (Bio-Rad). Quantification was done in the following manner: the area under the curve (AUC) of the bands was selected using the ‘Lane Profile’ tab and the adjusted volumes were taken. The adjusted volumes were then corrected for the amount of protein in each lane, using the adjusted total lane volumes of the Coomassie stained gels. The volume of the band at 55 kDa in the molecular weight marker (PageRuler Plus) was set to 100 (%), and the other bands were normalized accordingly. Further data analysis and statistics were carried out using Graphpad Prism. All given percentages are the mean values ± SEM of three individual experiments. Statistical analysis was performed using a one- or two-way ANOVA test with multiple comparisons (***p < 0.001; **p < 0.01; ns = not significant).

### Confocal Microscopy

CHOhA_3_AR and CHO-K1 cells were cultured in a 96-well plate. Upon reaching a confluency of about 90%, the medium was replaced by medium containing PSB-11 (final concentration: 1 μM) or 1% DMSO (control) and the cells were pre-incubated for 30 min (37 °C and 5% CO_2_). The medium was then replaced with medium containing **9** (final concentration: 50 nM) or 1% DMSO (vehicle), and the cells were incubated for 60 min (37 °C and 5% CO_2_). The cells were washed with PBS to remove unbound probe and afterward fixed using a 4% PFA in 10% formalin solution (15 min, rt). The remaining fixative was removed by washing with PBS and subsequently with 20 mM glycine in PBS. The cells were permeabilized by incubation in 0.1% saponin in PBS (10 min, rt). Remaining saponin was removed by a PBS wash, and the cells were stored at 4 °C until further usage. At the day of imaging, PBS was removed and the cells were incubated for 1 h in freshly prepared click mix (100 μL 100 mM CuSO_4_ in MilliQ, 100 μL 1 M NaAsc in MilliQ, 100 μL mM THPTA in MilliQ, 9.66 mL HEPES pH 7.4, and 40 μL 1 mM TAMRA-N_3_ in DMSO, added in the respective order). The remaining click mix was removed by washing with PBS, incubating for 30 min with 1% BSA in PBS and again washing with PBS. Cells were stored in 300 nM DAPI in PBS prior to imaging. Microscopy was performed on a Nikon Eclipse Ti2 C2 + confocal microscope (Nikon, Amsterdam, The Netherlands), and this system included an automated xy-stage, an integrated Perfect Focus System (PFS), and 408 and 561 nm lasers. The system was controlled by Nikon’s NIS software. All images were acquired using a 20× objective with 0.75 NA at a resolution of 1024 × 1024 pixels. The acquisition of 9 fields of view per well was done automatically using the NIS Jobs functionality. Representative images are shown in the figure and were created by using OMERO.^57^

### Data Analysis of Confocal Microscopy Experiments

CellProfiler (version 2.2.0) was used to create a binary image of the DAPI channel and to propagate the cytoplasmic area based on the DAPI binary. An overlay of the binary cytoplasm/TAMRA channel was generated to quantify per segmented pixel the TAMRA intensity. The sum of these intensities in the cytoplasm mask is referred to as the integrated TAMRA intensity in the cytoplasm. Segmentation results were further processed using Excel while GraphPadPrism 9 was used for data visualization and statistics.

### Click Reaction between **9** (LUF7960) and Cy5-N_3_

The click mix was freshly prepared by combining in a 2 mL Eppendorf tube 250 μL of 100 mM CuSO_4_ in MilliQ, 150 μL of 1 M NaAsc in MilliQ, 50 μL of 100 mM THPTA in MilliQ, and 50 μL of 50 μM Cy5-N_3_ in DMSO, in the same respective order. Next, 500 μL of 2 μM **9** (LUF7960) in 1% DMSO in MilliQ was added and the mixture was incubated for 1 h at rt while shaking (1000 rpm). The mixture was desalted using a Waters Sep-Pak C18 column (WAT054945). Briefly, the mixture was loaded on the column and washed three times with 1 mL of MilliQ. The product (**9**-Cy5) was then eluted using 1 mL of acetonitrile. The solvent was removed using an Eppendorf Concentrator Plus, and the residue was dissolved in DMSO to obtain the stock concentrations of **9**-Cy5.

### Flow Cytometry Experiments Using CHO Cells

CHOhA_3_AR and CHO-K1 cells were cultured as mentioned above. The cells were detached using Trypsin and centrifuged (5 min, 1500 rpm). The pellet was dissolved in medium (1 mL), the cells were counted, and the solution was diluted to 1,000,000 cells/mL. 100 μL of cell solution was added to each well of a 96-well plate. The plate was centrifuged (5 min, 1500 rpm), and the medium was replaced by medium containing PSB-11 (final concentration: 1 μM) or 1% DMSO (control). Cells were resuspended and incubated for 30 min (37 °C, 5% CO_2_). The plate was centrifuged (5 min, 1500 rpm), and the medium was replaced by medium containing pre-clicked **9**-Cy5 (final concentration: 50 nM). Cells were resuspended and incubated for 1 h (37 °C, 5% CO_2_). The plate was centrifuged (5 min, 1500 rpm), the medium was removed, and 1% BSA in PBS containing rabbitαhA_3_AR PE conjugate (1:500) was added. Cells were resuspended and incubated for 30 min at 4 °C. The cells were washed with 1% BSA in PBS to remove unbound probe and antibody and resuspended in 1% BSA in PBS. Cells were measured using a Cytoflex S (Beckman and Coulter, USA), and data was analyzed with FlowJo v10.0.7 (BD Life Sciences). The gating strategy is shown in Figure S2.

### Flow Cytometry Experiments Using Human Granulocytes

100 μL of PMNs or purified eosinophils (2·10^6^ cells/mL) in HEPES buffer was incubated with 50 μL of PSB-11 (final concentration: 1 μM) or 1% DMSO (control) in HEPES buffer for 30 min at rt. 50 μL of pre-clicked **9**-Cy5 (final concentration: 50 nM) or 1% DMSO (vehicle) was added, and the samples were incubated for 1 h at rt. The samples were then added to a 96 well-plate and centrifuged (5 min, 1500 rpm, 4 °C). The supernatant was removed, and the pellet was suspended in a solution containing antibody (Siglec-8 PE conjugate 1:100) in 0.5% HSA in PBS. The samples were incubated for 30 min at 4 °C, centrifuged (5 min, 1500 rpm, 4 °C), and washed once with 0.5% HSA in PBS. The final pellet was suspended in 0.5% HSA in PBS. Cells were measured on a Canto II flow cytometer (BD, Franklin Lakes, NJ, USA) and analyzed with FACSDiva software. The gating strategy is shown in Figure S2.

### Data Analysis of Flow Cytometry Experiments

Data analysis was performed using Graphpad Prism. MFI values shown are mean ± SEM from three individual experiments performed in duplicate (Figure 7A) or four individual experiments (Figure 7C). Statistical analysis was performed using a one-way ANOVA test with multiple comparisons (****p < 0.0001; ***p < 0.001; **p < 0.01; ns = not significant).

## Supporting Information Available

Additional figures, NMR spectra and HPLC traces (PDF)
Compounds **5**, **9**, and **13** docked in the Alphafold predicted model of the hA_3_AR (PDB, PDB, PDB)
Molecular formula strings with bioactivity data (CSV)

## Supplementary Material

jm3c00854_si_001.pdf

jm3c00854_si_002.pdb

jm3c00854_si_003.pdb

jm3c00854_si_004.pdb

## Abbreviations

A_1_AR: adenosine A_1_ receptor
A_2A_AR: adenosine A_2A_ receptor
A_2B_AR: adenosine A_2B_ receptor
A_3_AR: adenosine A_3_ receptor
ADA: adenosine deaminase
AfBP: affinity-based probe
AR: adenosine receptor
BCA: bicinchoninic acid
DAPI: 4′,6-diamidino-2-phenylindole
CHO: Chinese hamster ovary
CBB: Coomassie brilliant blue
CPA: N^6^-cyclopentyladenosine
CuAAC: copper-catalyzed alkyne–azide cycloaddition
DIPEA: *N*,*N*-diisopropylethylamine
EDC: 1-ethyl-3-(3-dimethylaminopropyl)carbodiimide
hA_1_AR: human adenosine A_1_ receptor
hA_2A_AR: human adenosine A_2A_ receptor
hA_2B_AR: human adenosine A_2B_ receptor
hA_3_AR: human adenosine A_3_ receptor
HRP: horseradish peroxidase
MFI: mean fluorescence intensity
NaAsc: sodium ascorbate
NECA: 5′-(*N*-ethylcarboxamido)adenosine
PMN: polymorphonuclear neutrophil
PNGase: peptide:*N*-glycosidase
PPI: protein–protein interaction
PTM: post-translational modification
TAMRA: carboxytetramethylrhodamine
THPTA: tris(3-hydroxypropyltriazolylmethyl)amine
