# Supplementary material for: Development of an Affinity-Based Probe to Profile Endogenous Human Adenosine A3 Receptor Expression
Source: J Med Chem. 2023 Aug 2;66(16):11399–413. doi: 10.1021/acs.jmedchem.3c00854 (PMC10461224; doi:10.1021/acs.jmedchem.3c00854)
Supplement: Supplementary file 1 — jm3c00854_si_001.pdf [file jm3c00854_si_001.pdf]

# Supporting Information

# Development of an Affinity-Based Probe to profile Endogenous Human Adenosine A3 Receptor Expression

Bert L. H. Beerkens, Inge M. Snijders, Joep Snoeck, Rongfang Liu, Anton T. J. Tool, Sylvia E. Le Dévédec, Willem Jespers, Taco W. Kuijpers, Gerard J.P. van Westen, Laura H. Heitman, Adriaan P. IJzerman, and Daan van der Es*

[1] Division of Drug Discovery and Safety, Leiden Academic Centre for Drug Research, Leiden University, Einsteinweg 55, 2333 CC Leiden, The Netherlands, E-mail: d.van.der.es@lacdr.leidenuniv.nl

[2] Department of Molecular Hematology, Sanquin Research, Plesmalaan 125, 1066 CX Amsterdam, The Netherlands

[3] Department of Pediatric Immunology, Rheumatology and Infectious Diseases, Emma Children's Hospital, Academic Medical Center, University of Amsterdam, Amsterdam, The Netherlands, Meibergdreef 9, 1105 AZ Amsterdam, The Netherlands

[4] Oncode Institute, Einsteinweg 55, 2333 CC, Leiden, The Netherlands

*Corresponding author, email: d.van.der.es@lacdr.leidenuniv.nl

**Contents**

Supporting Tables S2

Supporting Figures S4

NMR spectra S6

HPLC spectra S12

## Supporting Tables

**Table S1. Time-dependent apparent affinity of the synthesized AfBPs towards the $hA_1AR$.**

| Compound | pKi (pre-0h)[a] | pKi (pre-4h)[b] |
|---|---|---|
| **5** (LUF7930) | 5.28 ± 0.03 | 6.40 ± 0.14 |
| **9** (LUF7960) | 6.04 ± 0.03 | 7.14 ± 0.02 |
| **13** (LUF7934) | 6.32 ± 0.11 | 7.18 ± 0.06 |

[a] Apparent affinity determined from displacement of specific [$^3$H]DPCPX binding on CHO cell membranes stably expressing the $hA_1AR$ at 25 °C after 0.5 h of co-incubating probe and radioligand. [b] Apparent affinity determined from displacement of specific [$^3$H]DPCPX binding on CHO cell membranes stably expressing the $hA_1AR$ at 25 °C after 4 h of pre-incubation with the respective probe, followed by an additional 0.5 h of co-incubation with radioligand. Data represent the mean ± SEM of three individual experiments performed in duplicate.

**Table S2. Binding affinities of pre-clicked 9-Cy5 towards the $hA_3AR$ and $hA_1AR$.**

| Compound | $A_3AR$[a] | $A_1AR$[b] |
|---|---|---|
| **9**-Cy5 | 53% | 16% |

[a] % specific [$^3$H]PSB-11 displacement by 1 μM of respective probe on CHO cell membranes stably expressing the $hA_3AR$. [b] % specific [$^3$H]DPCPX displacement by 1 μM of respective probe on CHO cell membranes stably expressing the $hA_1AR$. Probes were co-incubated with radioligand for 30 min at 25 °C. Data represent the mean of two individual experiments performed in triplicate.

## Supporting Figures

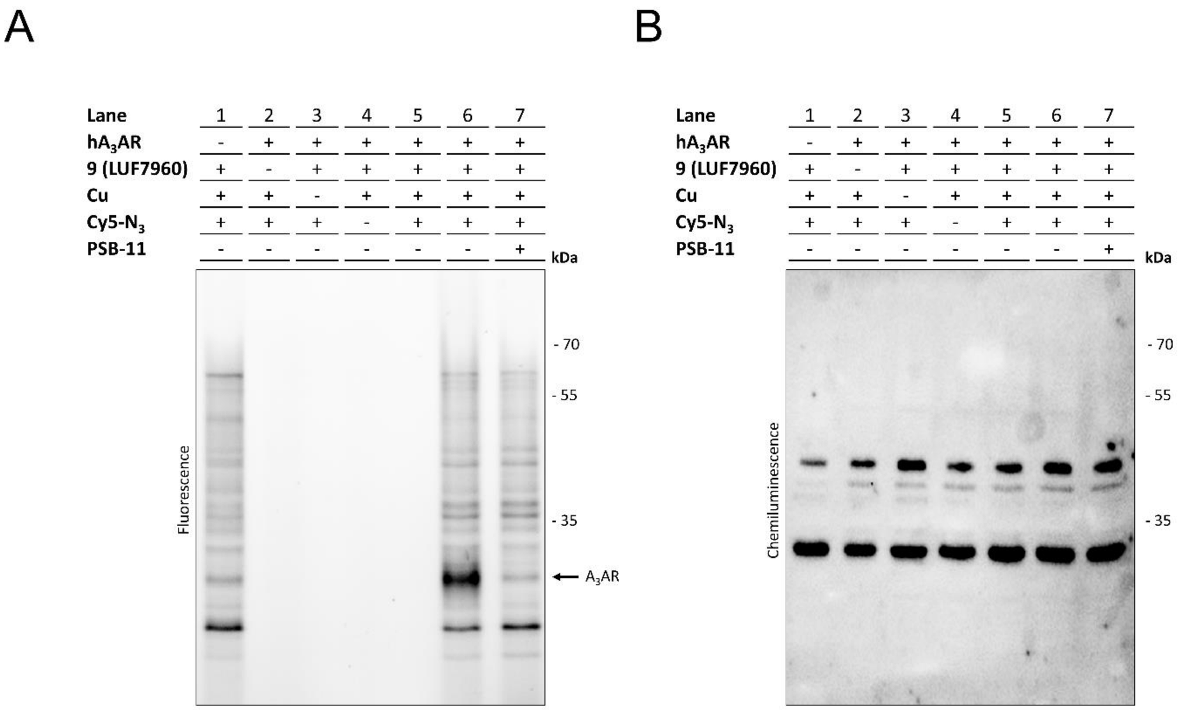


**Figure S1.** Labelling of the hA3AR is evident in SDS-PAGE, but not in western blot experiments. (A) A specific hA3AR band (lane 6) is observed upon measuring in-gel fluorescence after protein labelling by AfBP **9** (LUF7960); (B) No specific hA3AR band is observed upon measuring chemiluminescence after protein labelling by the commercially available antibody, i.e.: the labelled proteins appear in both membrane fractions with and without (lane 1) expression of the hA3AR. (A-B) Membrane fractions from CHO cells with and without (first lane) overexpression over the hA3AR were pre-incubated for 30 min with the selective antagonist PSB-11 or 1% DMSO (vehicle control), prior to incubation for 1 h with AfBP **9** (LUF7960). *N*-Glycans were removed using PNGase, probe-bound proteins clicked to Cy5-$N_3$, denatured using Laemmli buffer and subjected to SDS-PAGE. The gel was imaged using in-gel fluorescence (Figure A) and subsequently transferred to 0.2 μM PVDF blots (Bio-Rad) using a Bio-Rad Trans-Blot Turbo system (2.5 A, 7 min). The blot was blocked in 5% BSA in TBST (1 h, rt) and subjected to primary antibody (rabbitαhA3AR) 1: 10.000 in 1% BSA in TBST (overnight, 4 °C). The blot was washed (3 x TBST), subjected to secondary antibody (goatαrabbit-HRP) 1:2.000 in 1% BSA in TBST (1 h, rt), washed again (2 x TBST, 1 x TBS), activated with luminol enhancer and peroxide (3 min, rt, dark) and subsequently scanned on fluorescence (Figure A) and chemiluminescence (Figure B). Images are representatives of three individual experiments.

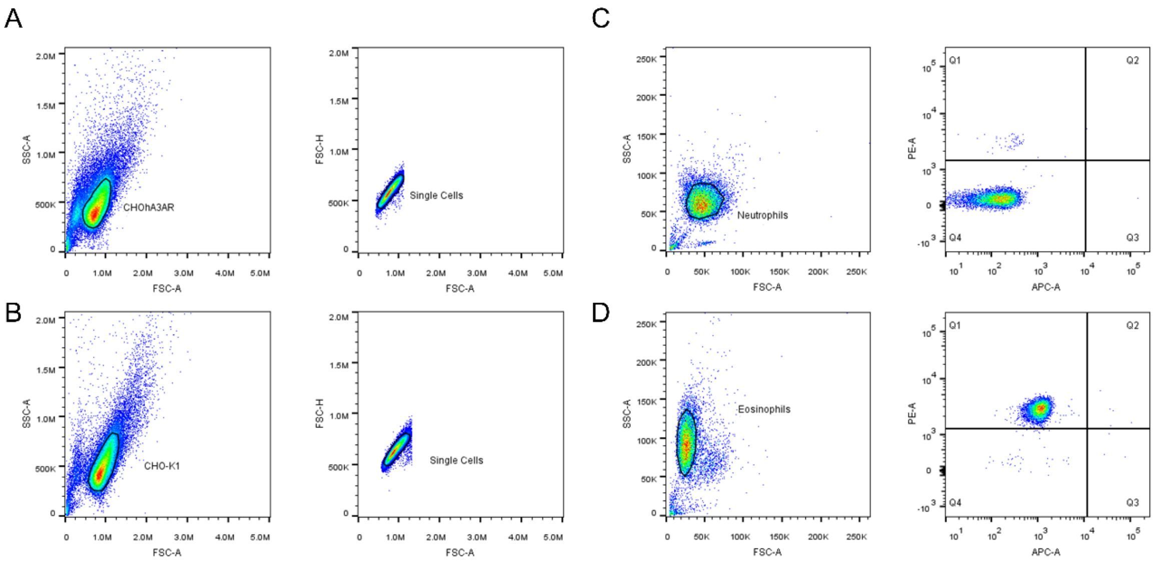


**Figure S2.** Gating strategy during analysis of the flow cytometry data. CHOhA$_3$AR (A) and CHO-K1 (B) cells were selected based on population density using SSC-A vs FSC-A gating and possible doublets were removed using FSC-H vs FSC-A gating. Neutrophils (C) and eosinophils (D) were selected based on population density using SSC-A vs FSC-A gating and PE signal of the PE-conjugated Siglec-8 antibody (selective marker for eosinophils) using PE-A vs APC-A gating. Q4 (neutrophils) and Q1 (eosinophils) were further analyzed on Cy5 intensity (APC channel).

**NMR spectra**

$^{1}$H NMR ((CD$_3$)$_2$SO, 500 MHz, 20 °C)

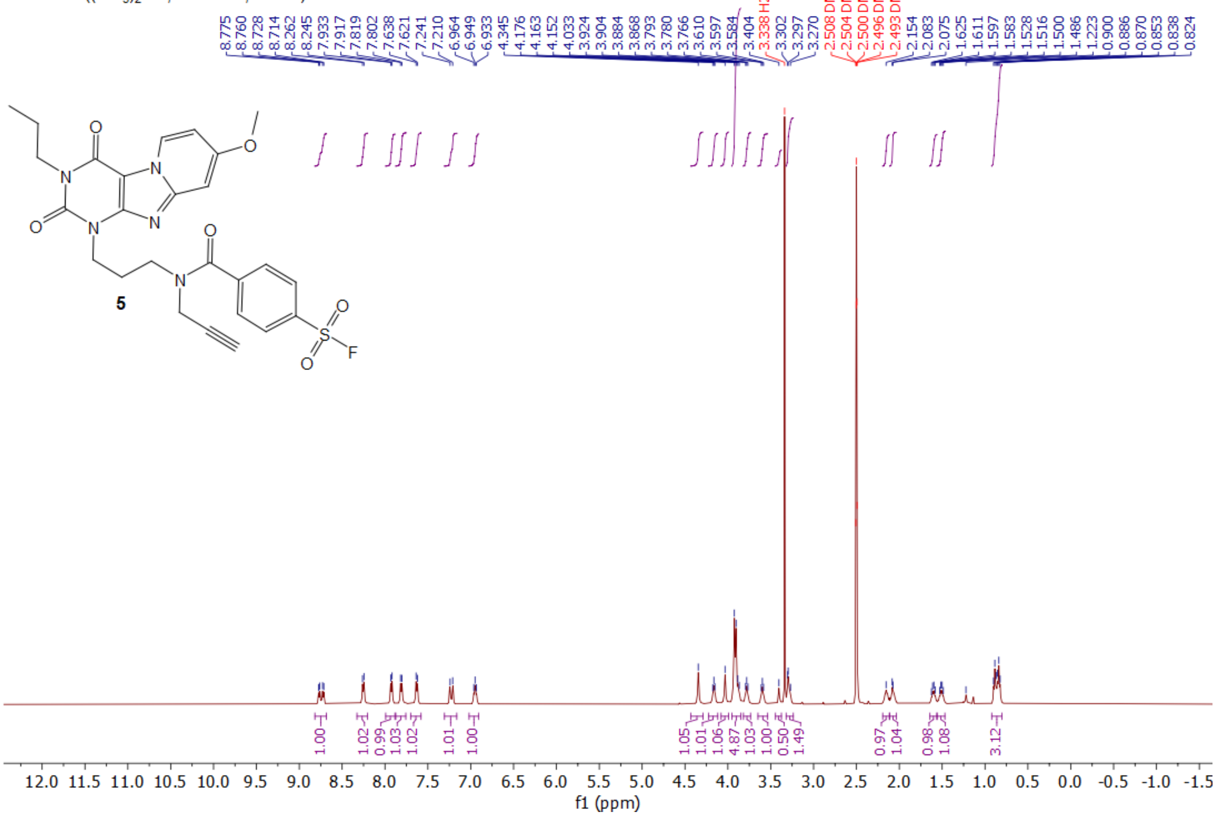


$^{1}$H NMR ((CD$_3$)$_2$SO, 500 MHz, 100 °C)

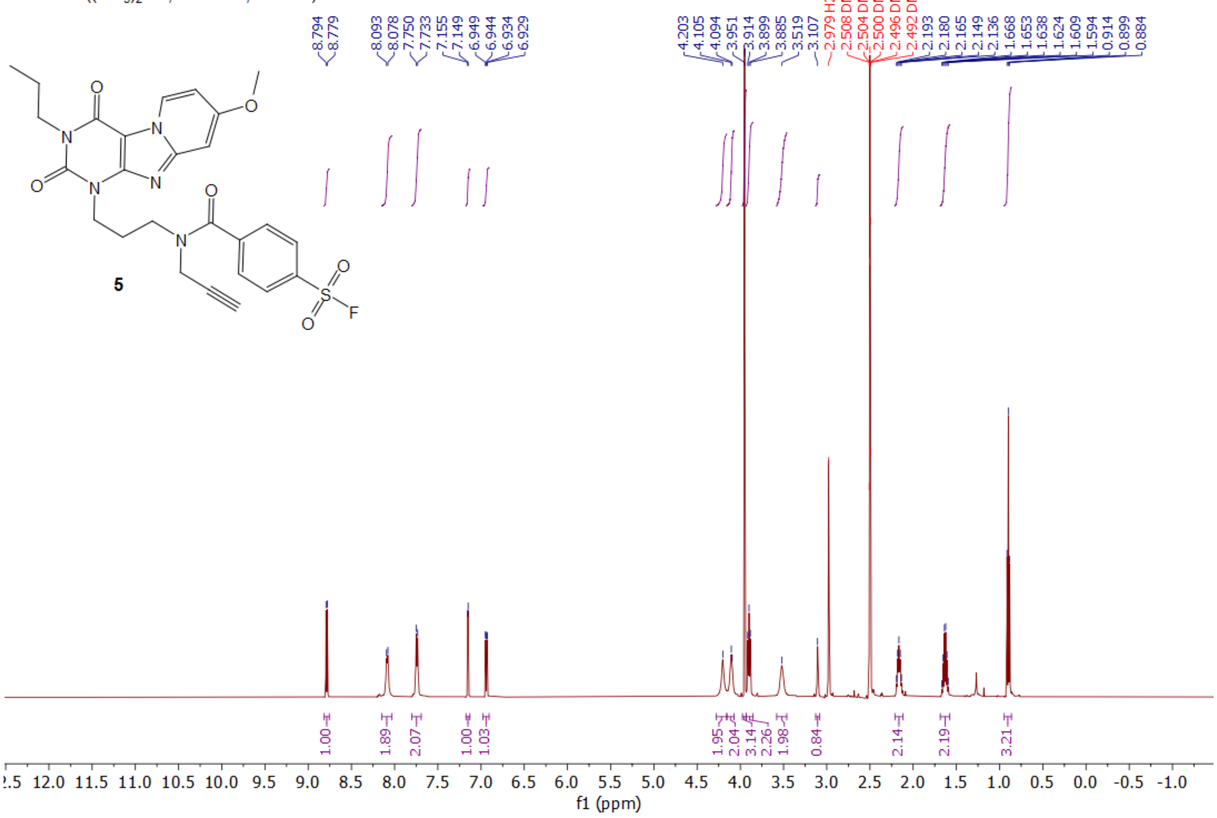

$^{13}C$ NMR ($(CD_3)_2SO$, 126 MHz, 20 °C)

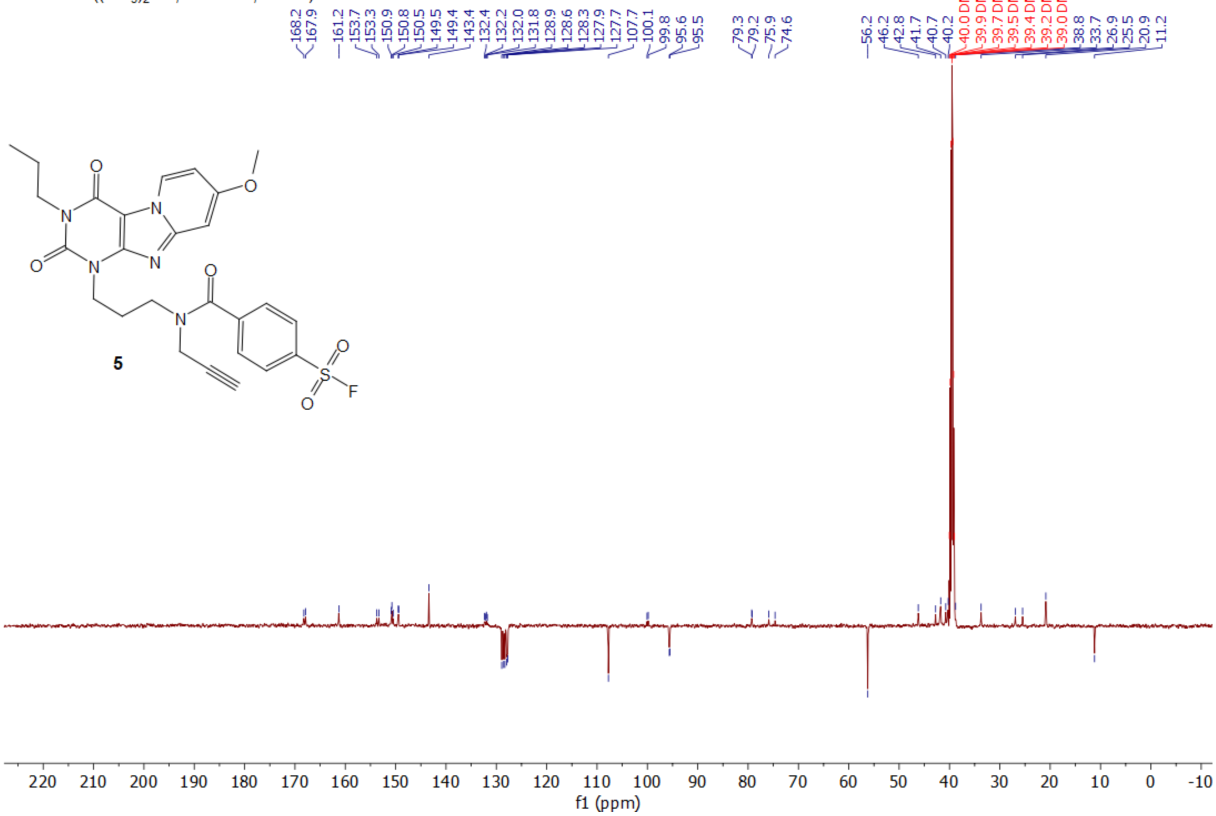


$^{13}C$ NMR ($(CD_3)_2SO$, 126 MHz, 100 °C)

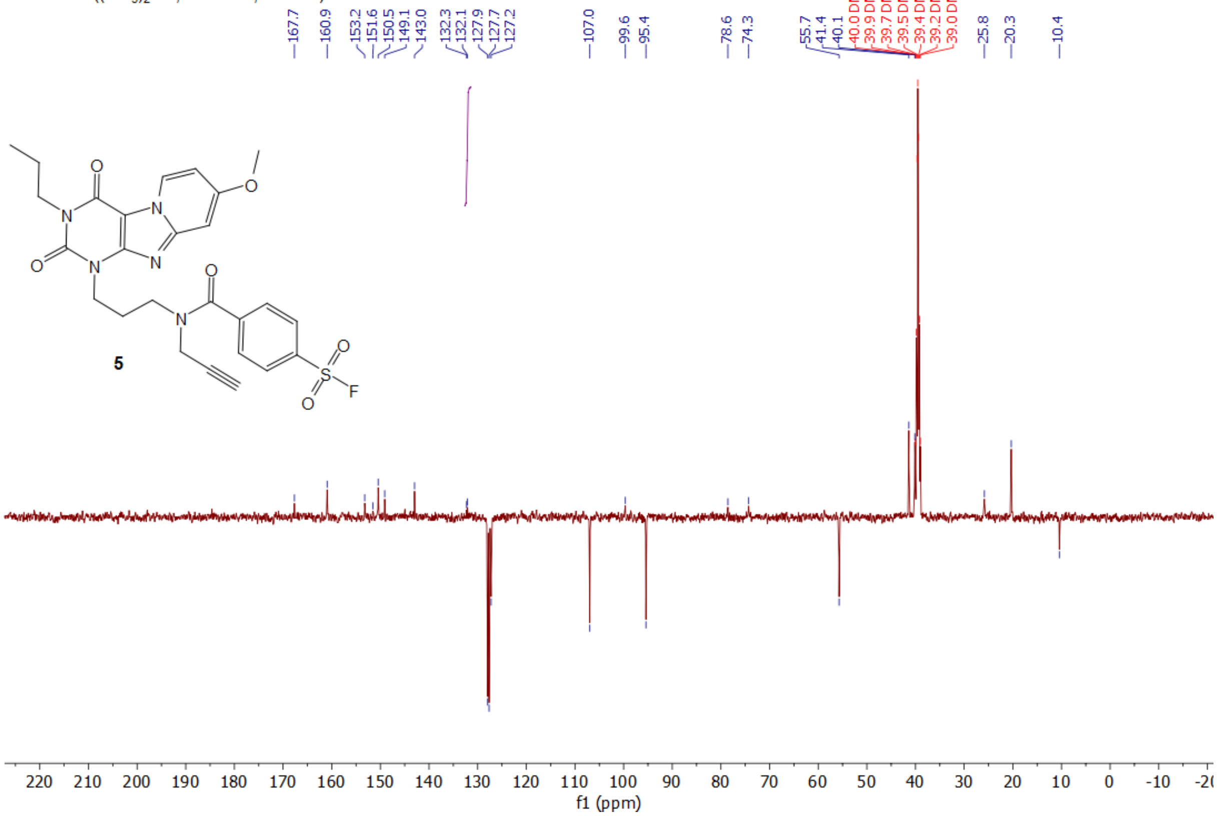

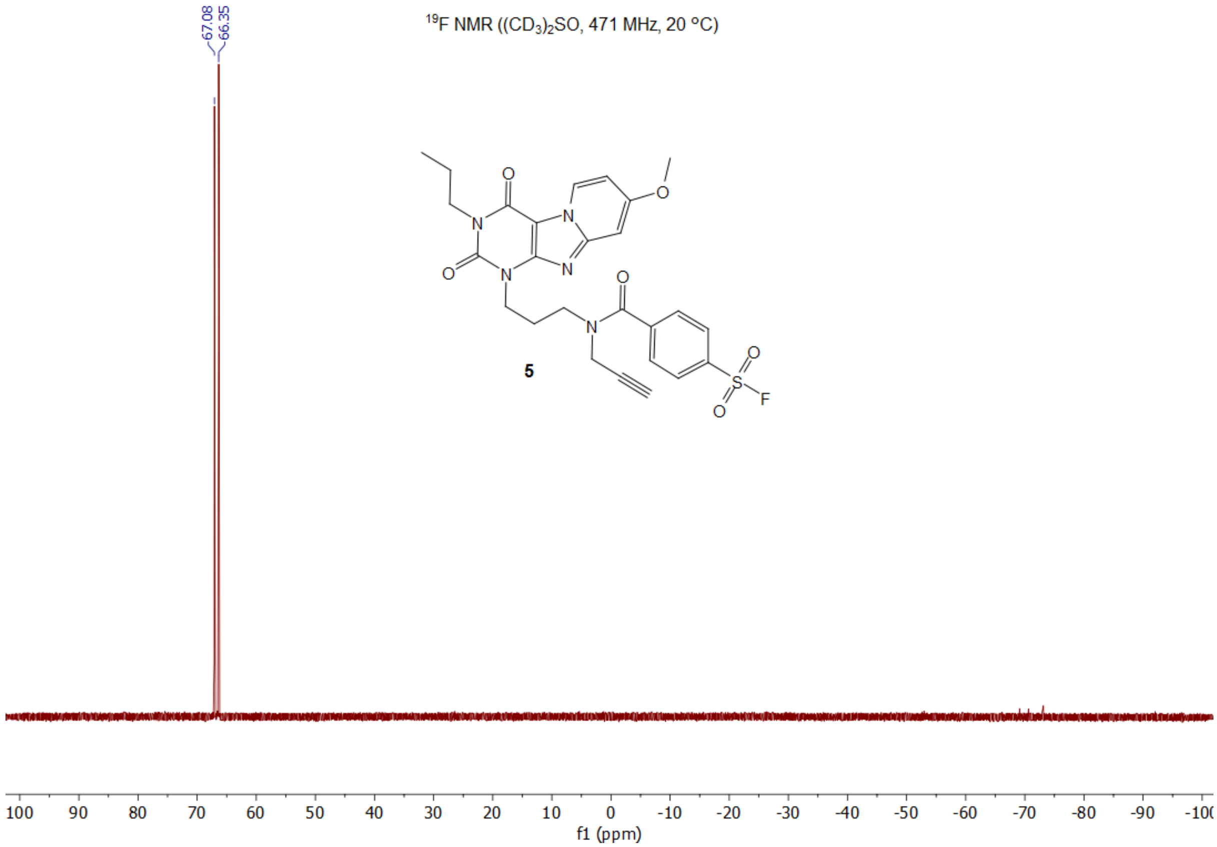

67.08
66.35
19F NMR ((CD3)2SO, 471 MHz, 20 °C)
5
100 90 80 70 60 50 40 30 20 10 0 -10 -20 -30 -40 -50 -60 -70 -80 -90 -100
f1 (ppm)


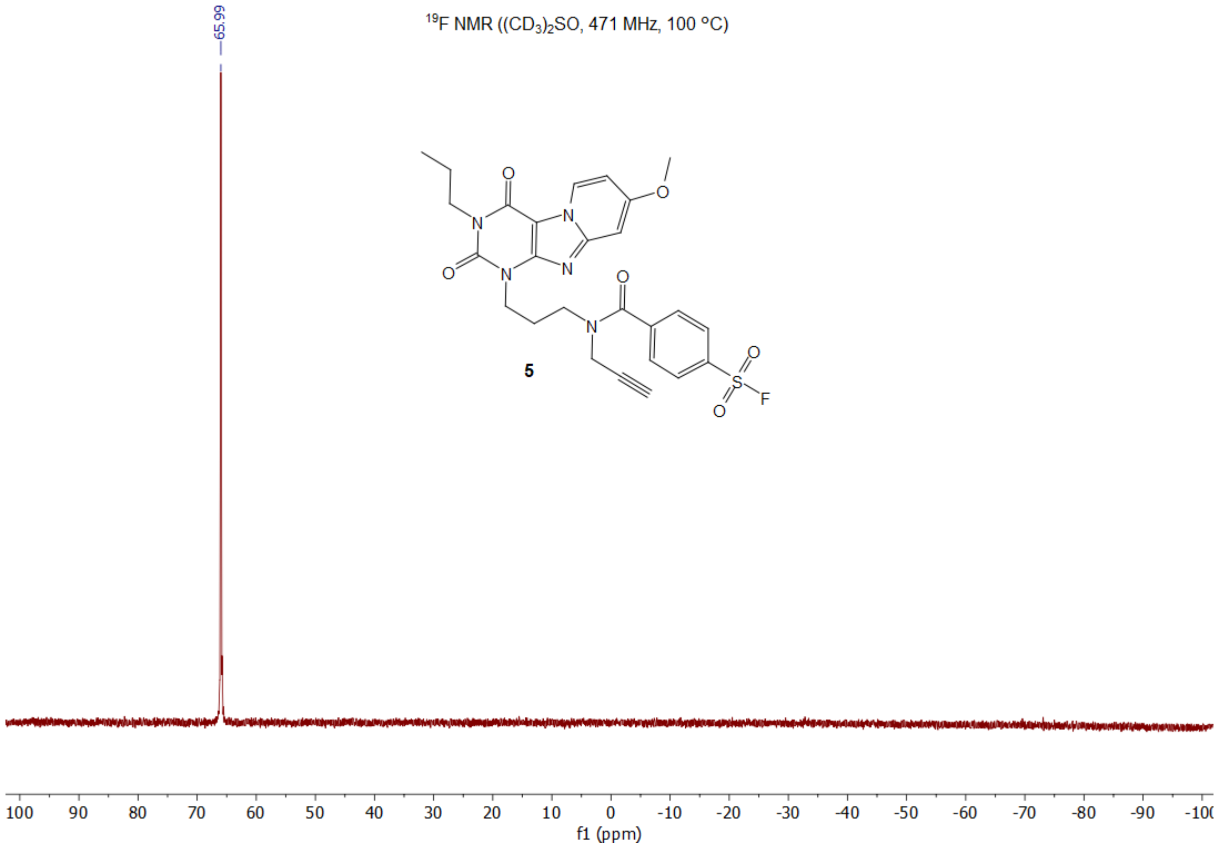

65.99
19F NMR ((CD3)2SO, 471 MHz, 100 °C)
5
100 90 80 70 60 50 40 30 20 10 0 -10 -20 -30 -40 -50 -60 -70 -80 -90 -100
f1 (ppm)

$^{1}H$ NMR ($CDCl_3$, 500 MHz)

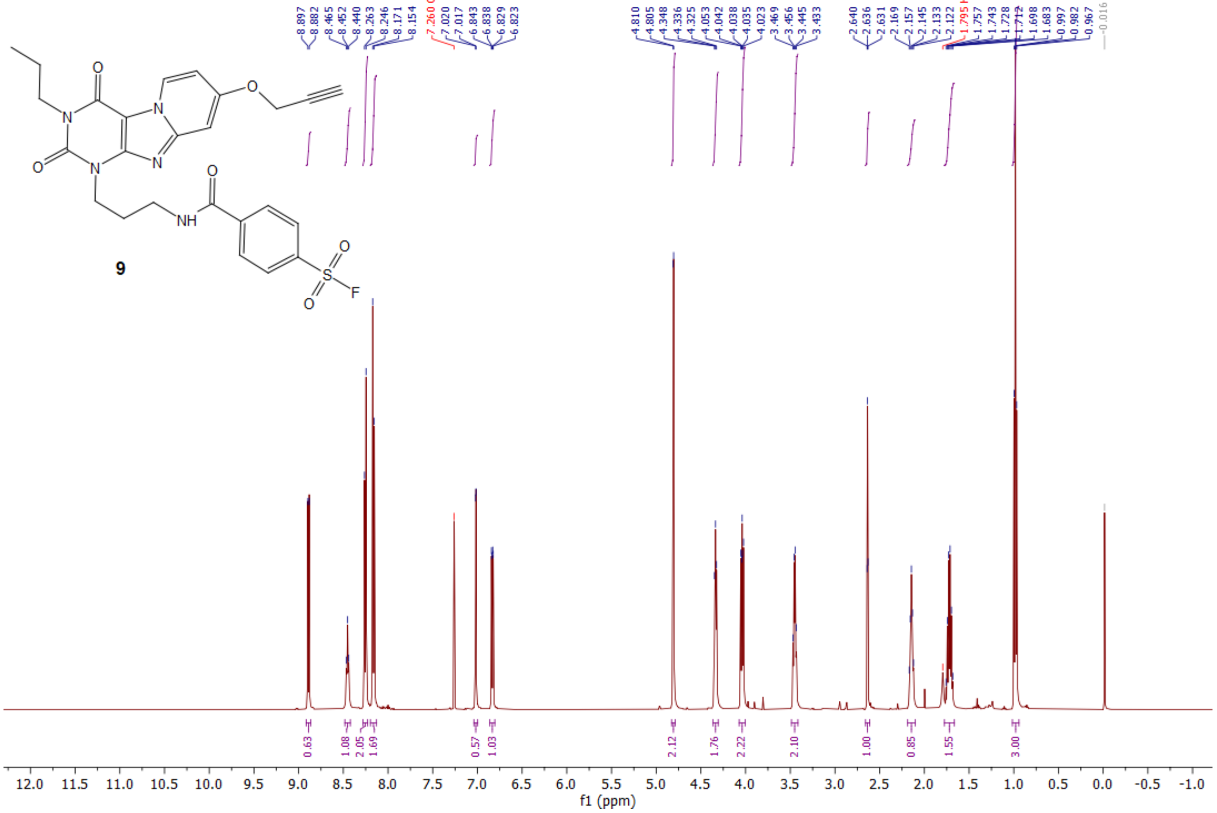


$^{13}C$ NMR ($CDCl_3$, 126 MHz)

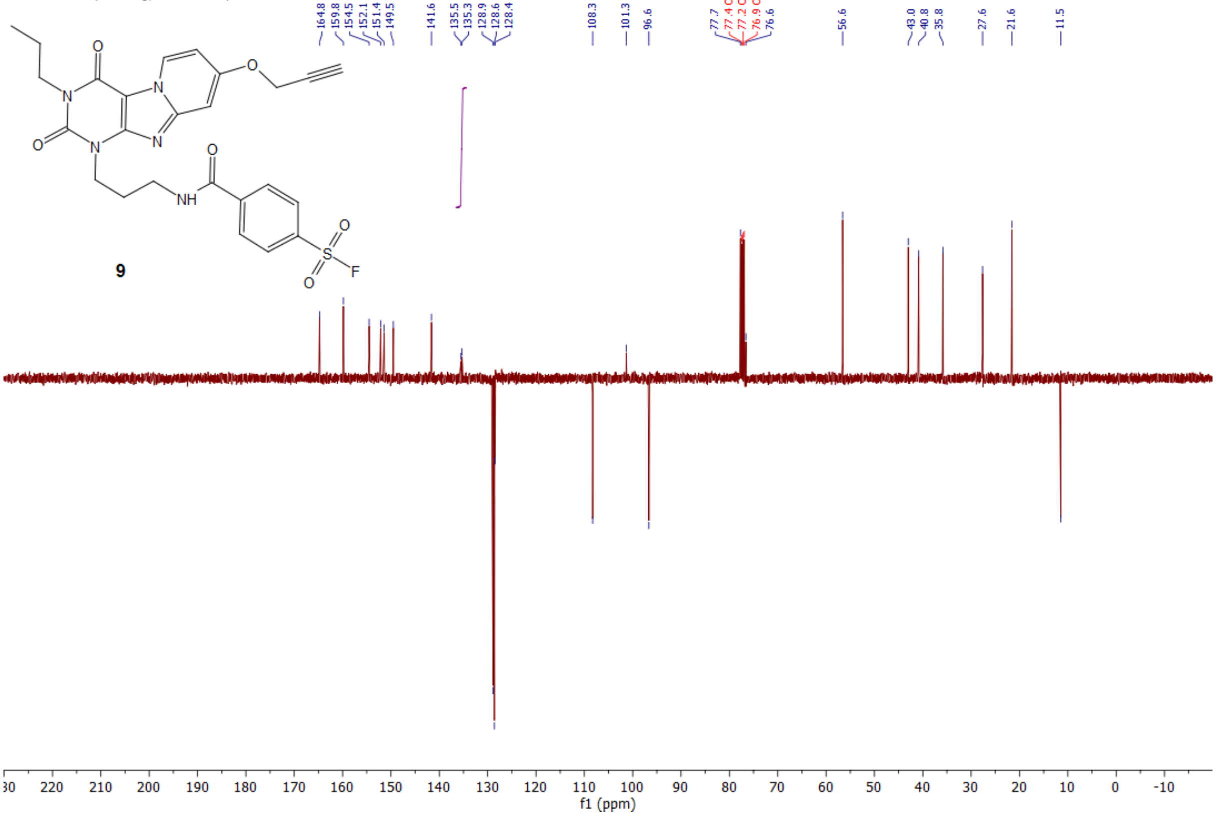

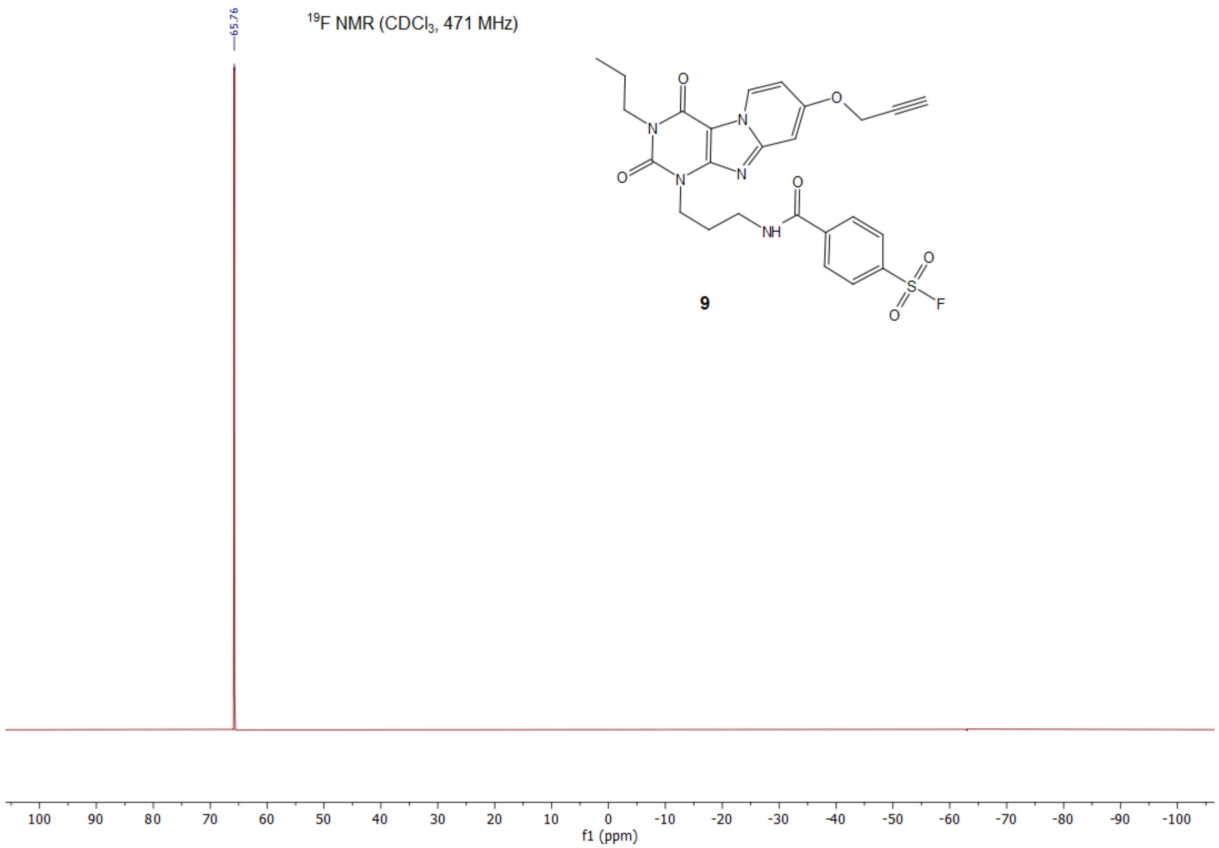

19F NMR (CDCl3, 471 MHz)
—65.76
9
f1 (ppm)


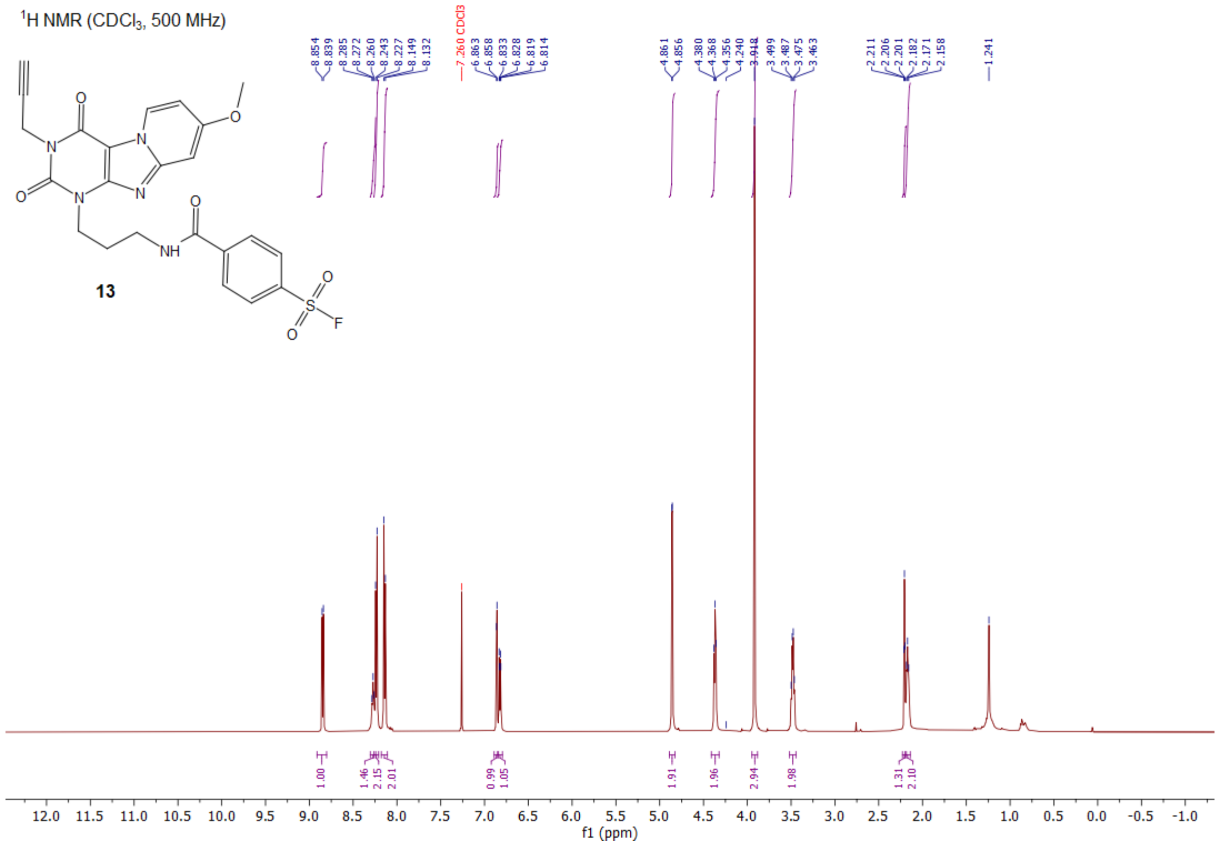

1H NMR (CDCl3, 500 MHz)
13
8.854 8.839 8.285 8.272 8.260 8.243 8.227 8.149 8.132
7.260 CDCl3
6.863 6.858 6.833 6.828 6.819 6.814
4.861 4.856 4.380 4.368 4.356 4.240 3.918 3.499 3.487 3.475 3.463
2.211 2.206 2.201 2.182 2.171 2.158
1.241
1.00 1.46 2.15 2.01 0.99 1.05 1.91 1.96 2.94 1.98 1.31 2.10
f1 (ppm)

$^{13}$C NMR ($CDCl_3$, 126 MHz)

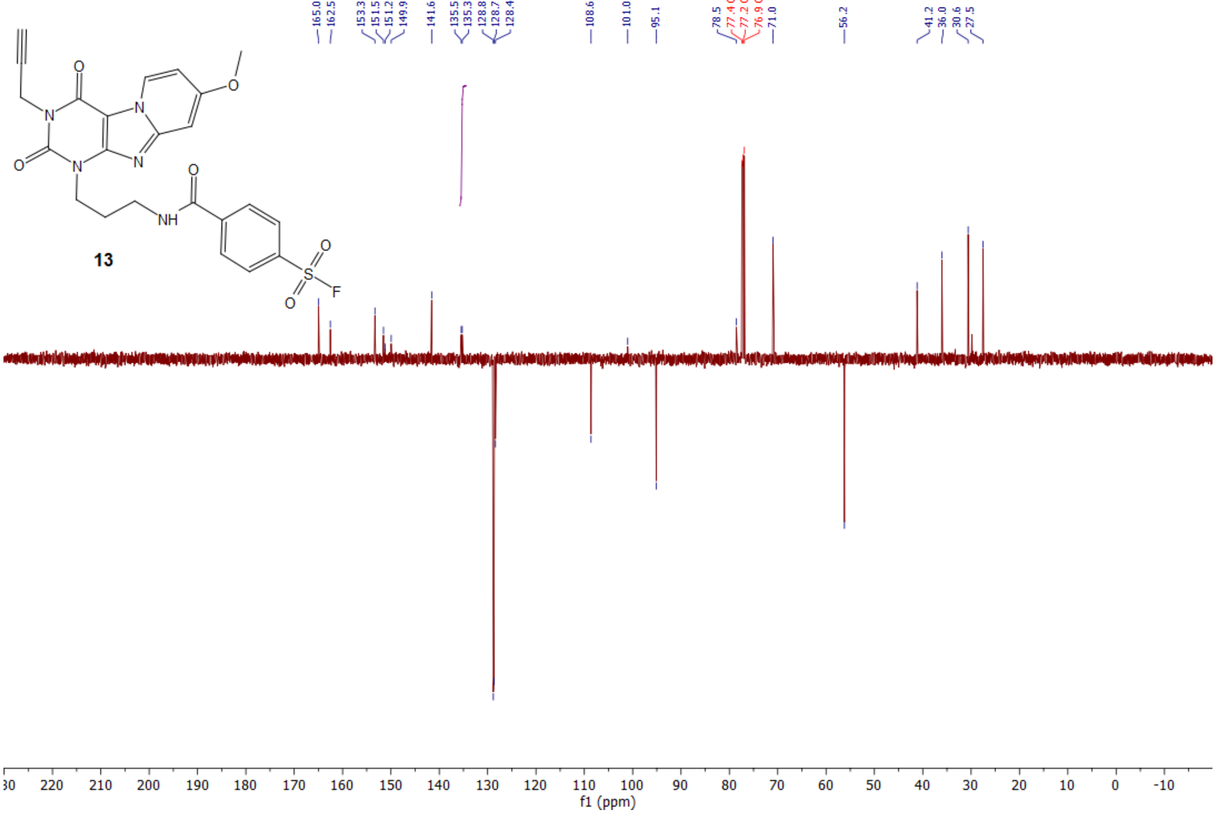


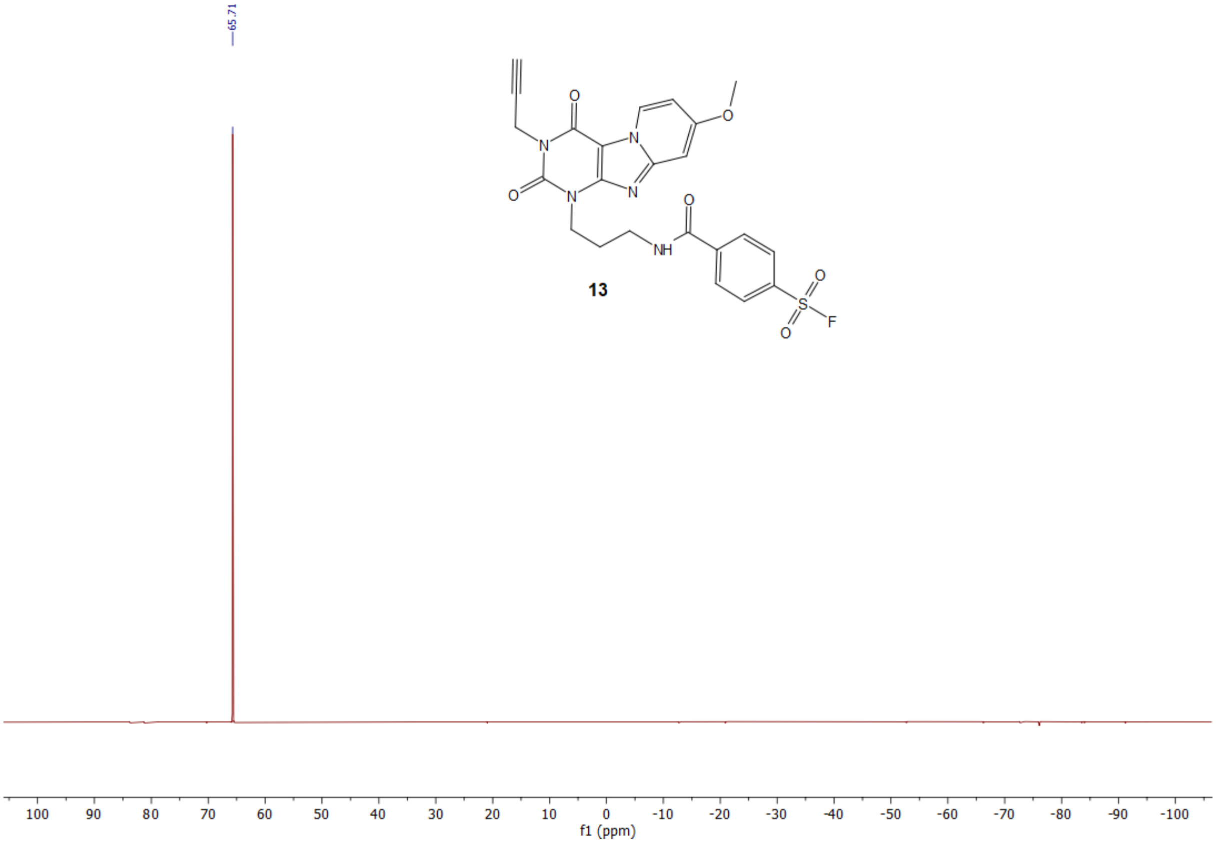

## HPLC spectra

HPLC traces compound **5**

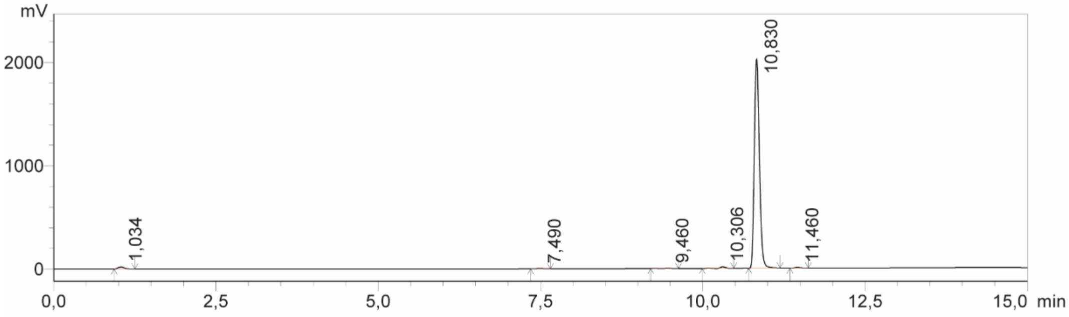


Peak Table

| Peak# | Ret. Time | Area | Height | Mark | Conc. | Area% |
|---|---|---|---|---|---|---|
| 1 | 1,034 | 137443 | 20151 | M | 1,201 | 1,201 |
| 2 | 7,490 | 23302 | 3725 | M | 0,204 | 0,204 |
| 3 | 9,460 | 48965 | 6086 | M | 0,428 | 0,428 |
| 4 | 10,306 | 109186 | 15700 | M | 0,954 | 0,954 |
| 5 | 10,830 | 11088587 | 2022031 | M | 96,896 | 96,896 |
| Total | | 11443776 | 2073619 | | 100,000 | 100,000 |

HPLC traces compound **9**

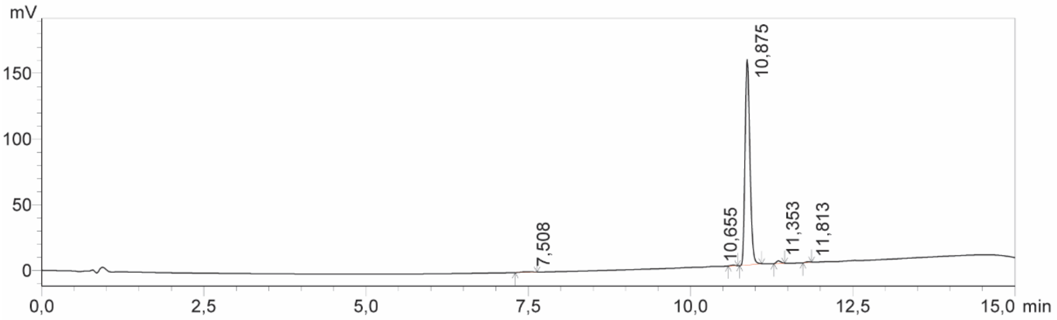


Peak Table

| Peak# | Ret. Time | Area | Height | Conc. | Area% |
|---|---|---|---|---|---|
| 1 | 7,508 | 4729 | 403 | 0,564 | 0,564 |
| 2 | 10,655 | 2425 | 504 | 0,289 | 0,289 |
| 3 | 10,875 | 819708 | 156575 | 97,702 | 97,702 |
| 4 | 11,353 | 9968 | 2073 | 1,188 | 1,188 |
| 5 | 11,813 | 2155 | 458 | 0,257 | 0,257 |
| Total | | 838985 | 160013 | 100,000 | 100,000 |

HPLC traces compound **13**

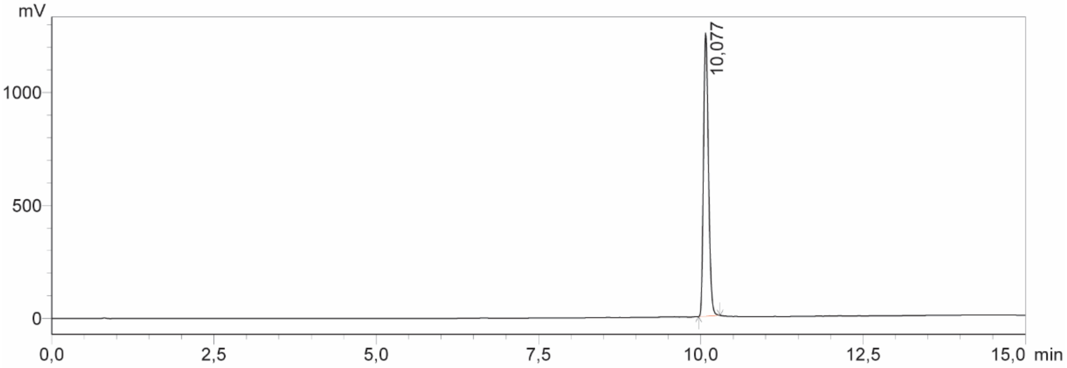


Peak Table

| Peak# | Ret. Time | Area | Height | Conc. |
|---|---|---|---|---|
| 1 | 10,077 | 6662256 | 1253802 | 100,000 |
| Total | | 6662256 | 1253802 | 100,000 |
